# When Two Become One: The Limits of Causality Analysis of Brain Dynamics

**DOI:** 10.1371/journal.pone.0032466

**Published:** 2012-03-16

**Authors:** Daniel Chicharro, Anders Ledberg

**Affiliations:** Center of Brain and Cognition, Department of Information and Communication Technologies, Universitat Pompeu Fabra, Barcelona, Spain; The University of Plymouth, United Kingdom

## Abstract

Biological systems often consist of multiple interacting subsystems, the brain being a prominent example. To understand the functions of such systems it is important to analyze if and how the subsystems interact and to describe the effect of these interactions. In this work we investigate the extent to which the cause-and-effect framework is applicable to such interacting subsystems. We base our work on a standard notion of causal effects and define a new concept called natural causal effect. This new concept takes into account that when studying interactions in biological systems, one is often not interested in the effect of perturbations that alter the dynamics. The interest is instead in how the causal connections participate in the generation of the observed natural dynamics. We identify the constraints on the structure of the causal connections that determine the existence of natural causal effects. In particular, we show that the influence of the causal connections on the natural dynamics of the system often cannot be analyzed in terms of the causal effect of one subsystem on another. Only when the causing subsystem is autonomous with respect to the rest can this interpretation be made. We note that subsystems in the brain are often bidirectionally connected, which means that interactions rarely should be quantified in terms of cause-and-effect. We furthermore introduce a framework for how natural causal effects can be characterized when they exist. Our work also has important consequences for the interpretation of other approaches commonly applied to study causality in the brain. Specifically, we discuss how the notion of natural causal effects can be combined with Granger causality and Dynamic Causal Modeling (DCM). Our results are generic and the concept of natural causal effects is relevant in all areas where the effects of interactions between subsystems are of interest.

## Introduction

Biological systems often consist of multiple interacting subsystems. An important step in the analysis of such systems is to uncover how the subsystems are functionally related and to study the effects of functional interactions in the system. A prominent example of a biological system with interacting subsystems is the brain, having interconnected ‘units’ at many different levels of description: e.g. neurons, microcircuits, and brain regions. It is the current belief that much of what we associate with brain function comes about through interactions between these different subsystems, and to characterize these interactions and their effects is one of the greatest challenges of the Neurosciences. Note that this is not only an experimental challenge but also a conceptual one. Indeed, even if given access to all the relevant variables in the nervous system, it is far from obvious how to analyze how brain activity relates to function.

In neurophysiology, the traditional approach to link brain activity to function is to perturb the nervous system and observe which variables change as a function of the perturbation. For example, many cells in primary sensory cortices elicit spikes at a rate that depends on a particular property of the stimulus (e.g. [1], [2]), and cells in the primary motor cortex tend to elicit spikes in relation to sensory conditioned movements (e.g. [3]). This approach is based on a conceptual cause-and-effect model: The perturbation (e.g. sensory stimulus) is the cause and the response of the nervous system (e.g. an increase in firing rate) the effect. There are two aspects of this experimental situation that allow a cause-and-effect interpretation: the perturbation is exogenous, or external, to the system; and, the effect follows the cause only after some temporal delay. That the perturbation is exogenous makes it possible to disentangle spurious dependencies from those due to a mechanistic (causal) coupling. The temporal delay between cause and effect is in line with the intuitive notion that a cause must precede its effect in time, and with our current understanding of the underlying mechanisms (e.g. how light is transformed into membrane currents and propagated through the visual system of the brain). This cause-and-effect model has been successfully applied to studies of response properties of single cells and brain regions, as well as to the relation between isolated limb movements and corresponding neuronal activity.

Perhaps inspired by the success of the cause-and-effect models in sensory and motor neurophysiology, workers have more recently started to look at interactions between different brain regions using the same framework. That is, researchers try to characterize the activity of one subsystem in terms of how it is caused by the activities of other subsystems. Indeed, a lot of theoretical and experimental work has been directed at investigating what has been called ‘direction of information flow’ (e.g. [4]), ‘causal relations’ (e.g. [5]), ‘causal influences’ (e.g. [6]), or ‘effective connectivity’ (e.g. [7], [8]), to just mention a few. A major difference with respect to the type of studies mentioned above is that the ‘cause’ is typically not a perturbation introduced by the experimenter. Rather, the joint natural activity of the subsystems is decomposed in causes and effects by statistical techniques. One crucial implication of this difference is that direction of the ‘causal flow’ is not restricted *a priori*: Contrary to the case of an externally applied stimulus, where the causality (if any) must ‘flow’ from the stimulus to the brain, in the case of two interacting brain systems it is quite possible that there is ‘causal flow’ in both directions. We will see that this bidirectionality has serious consequences for a cause-and-effect interpretation of interactions in the natural brain dynamics.

In this work we take a critical look at the cause-and-effect framework and demonstrate some fundamental shortcomings when this framework is used to study the natural dynamics of a system. We base our work on a standard model of causality that has emerged in the fields of statistics and artificial intelligence during the last decades (e.g. [9], [10]), synthesized in the framework of interventional causality proposed by Pearl [10]. In this framework external interventions (i.e. perturbations) of the system play a major role, both in defining the existence of causal connections between variables as well as in quantifying their effects. In our work we focus on situations where the interest is in characterizing the effects of natural interactions going on in a system. In such situations external interventions can not be used to quantify causal effects as they would typically disrupt the natural dynamics (c.f. [11]). We will analyze the conditions under which the natural interactions between subsystems can be interpreted according to a cause-and-effect framework. That is, our work is not about determining if a causal connection exists or not, but rather how to interpret the effects of existing causal connections. We derive conditions for when the interactions between two subsystems can be interpreted in terms of one system causing the other. The main requirement is that the causing subsystem acts as an exogenous source of activity. In particular, we show that the effects of interactions between mutually connected subsystems typically cannot be interpreted in terms of the effects that the individual subsystems exerts on the remaining ones. A conclusion of our work is therefore that the cause-and-effect framework is of limited use when characterizing the internal dynamics of the brain. The analysis is general and our conclusions therefore have important consequences for the interpretation of previous studies using different measures of ‘causality’, including Granger causality and Dynamic Causal Modeling.

## Results

In this section we first argue that it is important to distinguish between three different types of questions asked about causality and we introduce some basic notions about causal graphs. We then, in a number of subsections, develop what is needed to reach the main goal of the paper: to state conditions for when the effects of natural interactions between variables can be given a cause-and-effect interpretation. To reach this goal, we first give a brief overview of the interventional framework of causality. We then introduce an important distinction between situations where the main interest is the effect of external interventions and situations where the main interest is the impact of the causal connections on the dynamics happening naturally in the system. Next the conditions for when the natural interactions can be given a cause-and-effect interpretation are stated. Subsequently, we derive the consequences of these conditions for the special case of bivariate time series. We further suggest some novel approaches to the analysis of causal effects. Then we show how our work complements and extends two common approaches of ‘causality’ analysis: Granger Causality and Dynamic Causal Modeling (DCM). Finally we apply the analysis of causal effects to a simple model system to illustrate some of the theoretical points made.

In this work we are concerned with sets of variables and their interactions. We assume that the state of the variables is uncertain and that we have access to the (possibly time-dependent) joint probability over the variables. This is to avoid issues related to estimation from data. Our results are generic but it might be instructive to think of the variables as corresponding to the states of a set of neurons or other ‘units’ of the brain. We will further assume that the variables interact directly with each other, that is, that the variables are, or might be, causally connected. Note that experimental data sometimes reflect non-causal variables such as the blood oxygenation level depend (BOLD) signal and local field potentials (LFPs), in which case some additional level of modeling might be needed in order to make inferences about causality (c.f. [12]).

### Three questions about causality

To put our work in proper context and to facilitate a comparison with existing approaches to causality it is helpful to separate questions about causality into the following three types:

Q1: Is there a direct causal connection from 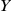 to 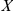
? (existence)
Q2: How is the causal connections from 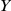 to 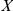 implemented? (mechanism)
Q3: What is the causal effect of 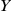 on 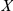
? (quantification)

(here 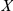 and 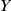 are two generic, and possibly high-dimensional, variables). We will show that it is important to keep these questions separate, and that different approaches are typically required to answer them. This might seem obvious, but in fact these three questions are often mixed into a ‘causality analysis’, and tools appropriate for the first two questions are often erroneously used to address also the third.

**Figure 1:**
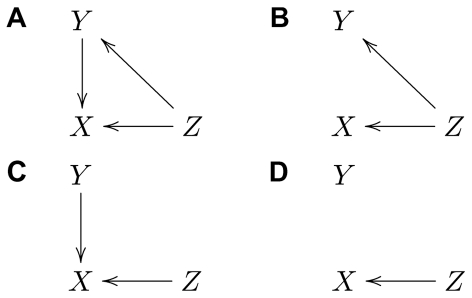
Causal graphs illustrating the effect of interventions. **A**: Graph showing a case where the statistical dependence between 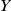 and 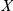 is (partly) due to a causal interaction from 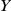 to 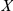. **B**: Graph showing a case where the statistical dependence between 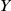 and 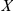 is induced solely by the confounding variable 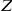. **C**: Graph corresponding to the intervention 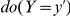 in the causal graph shown in **A**. **D**: Graph corresponding to the intervention 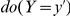 in the causal graph shown in **B**.

The first question (Q1) addresses the existence of a direct causal connection between two variables. A causal connection is a directed binary relation that carries only qualitative information. If the distribution of 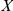 is invariant to perturbations in 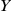 there is no causal connection from 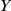 to 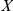. The total set of causal connections in a system is referred to as the *causal structure*. For a system containing a set 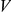 of variables 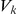, 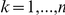, the causal structure can very conveniently be represented as a *causal graph* in which the nodes correspond to the variables and directed edges point from 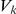 to 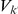 if there is a direct causal connection from 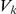 to 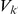. For example, Figure 1A, shows a causal graph where there are direct causal connections from 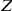 to 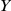, from 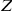 to 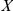, and from 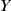 to 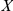. If the causal graph is without cycles (i.e. forming a directed acyclic graph, DAG) then the joint probability over the variables can be factorized according to the causal structure e.g. [10]. This is an important and useful result that we will use below and that is used extensively in the interventional framework of causality (see below). In fact this factorization following the causal structure is fundamental to relate the interventions to the joint probability (see Supporting Information S1). The causal graph contains all information needed to answer Q1. Unfortunately inferring the causal graph from observed data (here, the joint distribution) is in general not possible. A given joint distribution might be compatible with different causal structures [10], in which cases these graphs are said to be *observationally equivalent* [13]. Furthermore, the difficulty to infer the causal graph increases if there are hidden variables, i.e. variables that are not observed. The traditional approach to Q1 is therefore to experimentally modify (perturb) one variable and study the impact on the remaining ones. For example, the graphs in Figure 1A and 1B are both consistent with a statistical dependence between 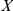 and 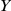. If 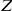 is not observed, it is not possible to distinguish between ‘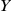 is causing 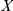
’ and ‘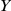 is not causing 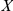
’ without intervening (i.e. perturbing) the system (see below). On the other hand, if 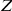 is observed we see that for the graph in Figure 1B, 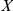 and 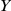 are conditionally independent given 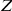, while for that in Figure 1A conditioning on 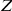 does not render 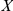 and 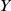 independent. In these types of causal structures 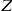 is considered a *confounder* because, being a common driver, it produces a statistical dependence between 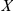 and 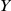 even without the existence of any direct causal connection between them.

Consider now the second question (Q2) about the mechanisms implementing the causal connections. In the ideal case the answer to this question would be given in terms of a biophysically realistic model of the system under study. However, often one has to make do with a phenomenological model that captures enough features of both structure and dynamics to give an adequate description of the system. That is, a model that is at some level functionally equivalent to the real physical system. Such a *functional model* would contain a formal description of how the variables in the system are generated, and how each variable depends on the other ones. For the causal graph of Figure 1A, the following formal equations define a functional model:
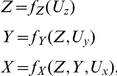



where the 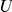-terms stand for random (non-observable) disturbances. We note that deriving a functional model from a detailed biophysical model, without modifying the impact of the causal interactions, might not be trivial. Nor is it trivial to go from experimental observations to a functional model that allows causal inference about the real physical system.

Since the model built to answer Q2 must already contain all the needed information to draw the corresponding causal graph, answering Q2 implies also answering Q1. In particular, any variable inside the function 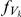 is considered a *parent* of 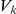 (the set of all parents is denoted 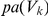) and an arrow from it to 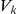 is included in the graph. Since the functional model is supposed to reflect the underlying mechanisms generating the variables, the parents 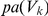 are the minimal set of variables with a direct causal connection to 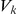. In such modeling approaches to causality it is important to emphasize that all notions of causality refer to the model and not to the system that is being modeled. In other words, only if the model is a faithful description of the process generating the variables can it be used as a model of real causal interactions.

In biology in general and in neuroscience in particular, the variables of interest are often functions of time. In such cases the functional model must be formulated in terms of dynamic equations (typically as difference or differential equations) and the variables represented in the causal graph will correspond to particular discrete times. In fact, for differential equations (i.e. representing a time-continuous dynamics), the causal graph corresponds only to a discrete representation of the equations (see [14] for a discussion of the correspondence between discrete and continuous models).

Since the answer to Q2 contains a model consistent with the mechanisms implementing the causal connections one may think that this would also be enough to answer Q3, i.e. the causal effect of 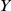 on 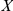. However, answering Q3 is not so straightforward. It is clear that the causal graph (Q1) does not contain information about the impact of the causal connections but only about their existence. Similarly, from a set of dynamic equations the resulting dynamics are only implicitly represented. This means that the effects of the causal connections modeled by the equations can typically not be read off directly from the equations. Rather, the quantification of the causal effect has to be done by analyzing the dynamics, either using observed data or, given the ‘correct’ model that generated the data, using simulated data from the model or analytical techniques. However, even if the required data are available, one further needs to clearly define what is meant by the *causal effect* that results from the causal connections. Therefore, question Q3 has to be addressed separately and is typically not reducible to answering Q2 or Q1.

In the rest of this work we will focus on this last question related to the analysis and quantification of causal effects (Q3). Following the distinction between the three questions about causality discussed above we will be very precise with our terminology: First, when talking about *causal connections* we will refer only to the causal structure in the graph. That is, a causal connection from variable 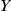 to variable 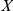 exists if and only if it is possible to go from 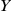 to 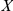 following a path composed by arrows whose direction is respected. In particular, a *direct causal connection* from 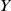 to 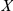 is equivalent to the existence of an arrow from 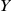 to 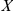. For two sets of variables the causal connection between the sets exists if it exists between at least a pair of variables. Second, when talking about the *causal effect* from 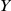 to 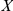 we will refer to the impact or influence of the causal connections from 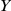 to 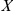. This impact depends on the causal structure, on the actual mechanisms that implement it, and is, as we will see, only appreciable in the dynamics.

### Causal effects in the framework of Pearl's interventions

In this section we will define causal effects using the framework of interventional causality developed by Judea Pearl and coworkers (e.g. [10]). We note that this framework is closely related to the potential outcomes approach to causality developed by Donald Rubin and coworkers (e.g. [9]) and that the causal effect used in that approach is fully compatible with the corresponding entity in the interventional framework. The definition of causal effects stated below can therefore be considered a ‘standard’ definition.

The starting point of the interventional framework is the realization that a causal connection between two variables can typically only be identified by intervening. That is, by *actively perturbing one variable and studying the effect on the other*. This is of course what experimentalists typically would do to study cause-and-effect relations. As a motivating example, consider paired recordings of membrane potentials of two, possibly interconnected, excitatory neurons (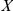 and 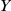 say). If we observe that membrane potential of neuron 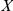 tends to depolarize briefly after neuron 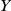 elicited an action potential, we might be tempted to conclude that 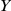 has causal influence over 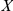. However, the same phenomenon could easily be accounted for by that 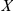 and 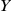 are receiving common (or at least highly correlated) inputs. The obvious thing to do in order to distinguish these two scenarios is to intervene, i.e. to force 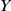 to emit an action potential (e.g. by injecting current into the cell). If depolarizations of the membrane potential of 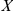 are consistently found after such interventions, we are clearly much more entitled to conclude that 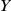 has a causal influence over 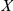. Note that the crucial aspect of the intervention is that it ‘forces’ one variable to take a particular value (e.g. fire an action potential) independently of the values of other variables in the system. It therefore ‘breaks’ interdependencies that are otherwise part of the system and hence can be used to distinguish between causal and spurious associations. The recent study by Ko et al. [15] is an excellent example of how interventions can be used to determine the causal structure between single neurons, and how this structure can account for observed statistical dependencies.

One of the contributions of the interventional framework of causality is that it formalizes the notion of an intervention and develops rules for how interventions can be incorporated into probability theory [10]. Symbolically an intervention is represented by the ‘
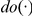
’ operator. For example, setting one variable, 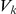, in the system to a particular value, 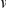, is denoted 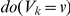. These interventions to a fixed value are commonly used in framework of Pearl's causality. Experimental interventions rarely can be exactly controlled, but the important point is not that a variable can be fixed to a given value but that the mechanisms generating this variable are perturbed. We will see below that the variability in the intervention can be captured introducing a probability distribution of interventions 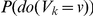. The effect of an intervention on the joint distribution is most easily seen when the joint distribution is factorized according to a causal graph. In this case the intervention 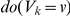 corresponds to deleting the term corresponding to 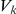 in the factorization and setting 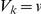 in all other terms depending on 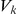. This truncated factorization of the joint distribution is referred to as the *postinterventional distribution*, that is, the distribution resulting from an intervention. In Supporting Information S1 we give a formal definition of the effect of an intervention and some examples. Graphically, the effect of an intervention is particularly illuminating: intervening in one variable corresponds to the removal of all the arrows pointing to that variable in the causal graph. This represents the crucial aspect of interventions mentioned above: intervention ‘disconnects’ the intervened variable from the rest of the system. Figure 1A and B illustrate two different scenarios for how a statistical dependence between 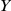 and 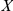 could come about. If only 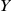 and 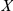 are observed, these scenarios are indistinguishable without intervening. The causal graphs corresponding to the intervention 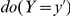 are shown in Figure 1C and D. It is clear that only the graph shown in Figure 1C implies a statistical relation between 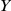 and 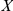 illustrating how the intervention helps to distinguish between causal and spurious (non-causal) associations. Indeed, interventions can generally be used to infer the existence of causal connections. Conditions and measures to infer causal connectivity from interventions have been studied for example in [16], [17].

Given this calculus of interventions, *the causal effect of the intervention of a variable 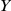 on a variable* 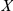 is defined as the postinterventional probability distribution 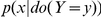 (see Definition 3.2.1 in [10] and Supporting Information S1). This definition understands the causal effect as a function from the space of 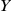 to the space of probability distributions of 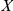. In particular, for each intervention 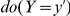, 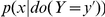 denotes the probability distribution of 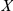 given this intervention. In Supporting Information S1 we show how 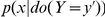 can be computed from a given factorization of the joint distribution of 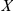 and 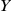. Note that this definition of causal effect is valid also if 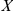 and 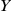 are multivariate.

This definition of causal effects is very general and in practice it is often desirable to condense this family of probability distributions (i.e. one distribution per intervention) to something lower-dimensional. Often the field of study will suggest a suitable measure of the causal effect. Consider the example of the two neurons introduced above, and let 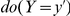 denote the intervention corresponding to making neuron 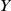 emit an action potential. To not introduce new notation we let 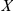 and 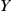 stand for both the identity of the neurons as well as their membrane potentials. Then a reasonable measure of the causal effect of 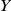 on 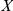 could be
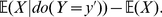
(1)


That is, the difference between the expected values of the postinterventional distribution 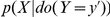 and the marginal distribution 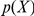 (c.f. [18]). In other words, the causal effect would be quantified as the mean depolarization induced in 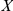 by an action potential in 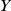. Clearly this measure does not capture all possible causal effects, for example, the variability of the membrane potential could certainly be affected by the intervention.

Intervening one variable is similar to conditioning on this variable, this is illustrated both in the notation and also in the effect of an intervention on the joint distribution. However, there is a very important difference in that an intervention actually changes the causal structure whereas conditioning does not. As mentioned above, it is this aspect of the intervention that makes it a key tool in causal analysis. Formally, this difference is expressed in that 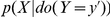 in general differs from 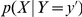. Consider for example the case when 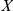 is causing 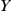 but not the other way around, i.e. 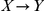, then 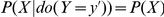 whereas, in general, 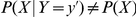.

A very important and useful aspect of this definition of casual effect is that if all the variables in the system are observed the causal effect can be computed from the joint distribution over the variables in the observed non-intervened system. That is, even if the causal effect is formulated in terms of interventions, we might not need to actually intervene in order to compute it. See Supporting Information S1 for details of this procedure and S2 for the calculation of causal effects in the graphs of Figure 1. On the other hand, if there are hidden (non-observed) variables, physical intervention is typically required to estimate the causal effect.

### Requirements for a definition of causal effect between neural systems

The definition of causal effects stated above is most useful when studying the effect of one or a few singular events in a system, that is, events isolated in time that can be thought of in terms of interventions. However, in neuroscience the interest is often in functional relations between different subsystems over an extended period of time (say, during one trial of some task). Furthermore, the main interest is not in the effect of perturbations, but in the interactions that are part of the brains natural dynamics. Consider for example the operant conditioning experiment, a very common paradigm in systems neuroscience. Here a subject is conditioned to express a particular behavior contingent upon the sensory stimuli received. Assume we record the simultaneous activity of many different functional ‘units’. Then a satisfactory notion of causal effect of one unit on another should quantify how much of the task-related activity in one unit can be accounted for by the impact of the causal connections from the other, and not the extent to which it would be changed by an externally imposed intervention. Of course, there are other cases where the effect of an intervention *is* the main interest, such as for example in studies of deep brain stimulation (e.g. [19]). In these cases the interventional framework is readily applicable and we will consequently not consider these cases further. We will instead focus on the analysis of natural brain dynamics which is also where DCM and Granger causality typically have been applied.

These considerations indicate some requirements for a definition of causal effects in the context of natural brain dynamics. First, causal effects should be assessed in relation to the dynamics of the neuronal activity. From a modeling point-of-view this implies that the casal effect can typically not be identified with parameters in the model. Second, the causal effects should characterize the *natural* dynamics, and not the dynamics that would result from an external intervention. This is because we want to learn the impact of the causal connections over the unaltered brain activity.

We will refer to causal effects that fulfill these requirements as *natural causal effects between dynamics*. We will now see that it is possible to derive a definition of natural causal effects between dynamics from the interventional definition of causal effects. We start by examining when natural causal effects between variables exist and in the following section we consider natural causal effects between dynamics.

### Natural causal effects

As explained above, a standard way to define causal effects is in terms of interventions. Yet, many of the most pertinent questions in neuroscience cannot be formulated in terms of interventions in a straightforward way. Indeed, workers are often interested in the ‘the influence one neural system exerts over another’ in the unperturbed (natural) state [8]. In this section we will state the conditions for when the impact of causal connections from one subsystem to another (as quantified by the conditional probability distribution) can be given such a cause-and-effect interpretation.

We first consider the causal effect of one isolated intervention. For a given value 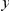 of the random variable 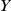, we define the *natural causal effect of 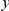 on the random variable* 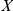 to be 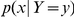 if and only if
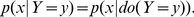
(2)


In words, if and only if conditioning on 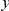 is identical to intervening to 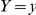, the influence of 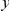 on 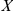 is a causal effect that we call a *natural causal effect*. Since the observed conditional distribution is equal to the postinterventional distribution given 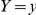, we interpret this as the intervention naturally occurring in the system. Note that this definition implies that if Eq. 2 does not hold, then the natural causal effect of 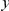 on 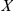 does not exist.

Next we formulate the natural causal effect between two (sets of) random variables. The natural causal effect of 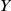 on 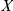 is given by
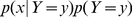
(3)


if and only if Eq. 2 holds for each value of 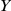. Note that Eq. 3 is a factorization of the joint distribution of 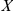 and 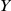. Indeed we have
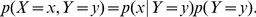
(4)


This means that if Eq. 2 holds for each value of 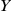 then the natural causal effect of 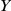 on 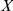 is given by the joint distribution of 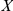 and 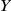. At first glance it might seem strange that the factor 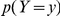 appears in the definition of the natural causal effect (Eq. 3). After all, the effects of the causal interactions are ‘felt’ only by 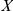, and we think of 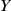 as being the cause. However, it is clear that the conditional distributions 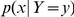 will in general depend on 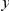 which means that to account for the causal effects of 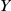 on 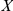 we need to consider all the different values of 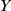 according to the distribution with which they are observed. This means that we must consider how often the different single natural interventions 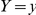 happen, that is, we need to include 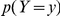 in the definition.

An important characteristic of natural causal effects is that the interventions they represent are not chosen by an experimenter (or policy maker) but are ‘chosen’ by the dynamics of the system itself. This means that we can think of the natural causal effect of 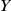 on 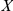 as the joint effect of all possible interventions 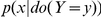 with the additional constraint that the distribution of the interventions is given by
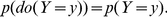
(5)


We can thus separate the definition of the natural causal effect from *variable* 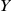 to 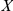 into two different criteria. The first one is a *criterion of existence of natural causal effects* of 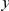 on 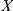 (Eq. 2), which determines when interventions occur naturally in the system. The second one, is a *criterion of maintenance of the natural joint distribution* (Eq. 5), which interprets the observed marginal distribution of the intervened variable as a distribution of interventions, so that the natural joint distribution is preserved (Eq. 4).

We now turn to the conditions on the causal structure under which natural causal effects exist. This means that we need to identify the conditions for which
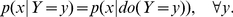
(6)


**Figure 2:**
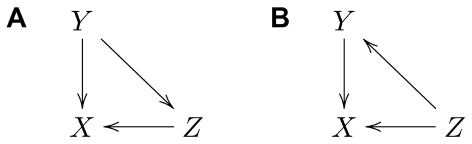
Natural causal effects. Examples of causal graphs illustrating when the effect of the influence of 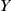 on 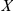 can be interpreted as a natural causal effect (**A**) and when it cannot (**B**).

In the interventional framework, this condition on the causal effect on 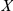 of intervening 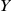 is called *not confounded* (Ch. 6 in[10]). Importantly, the fulfillment or not of this condition is determined only by the causal structure. In particular, in Supporting Information S1 we demonstrate that Eq. 6 holds if the following two conditions are fulfilled: First, that there are no causal connections in the opposite direction (i.e. from 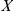 to 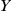). Second, that there is no common driver of 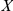 and 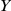. These two conditions assure that the dependence reflected in the conditional probability 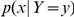 is specific for the causal flow from 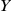 to 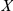. Notice though, that the presence of mediating variables is allowed, that is, a natural causal effect can be due to indirect causal connections. See Figure 2A for an illustration of a causal graph supporting natural causal effects that are partly indirect.

We emphasize that if Eq. 6 does not hold then the impact on 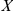 of the causal connections from 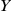 to 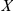 cannot be given a cause-and-effect interpretation. An example of this is given in Figure 2B where 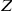 is a common driver of both 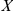 and 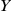 which therefore precludes a cause-and-effect interpretation of the joint distribution of 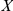 and 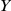. In this case we can still calculate the causal effect of an intervention, 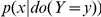, according to
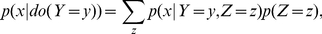
(7)


see Supporting Information S1 for a detailed calculation. It might seem contradictory that on the one hand side, the impact of the causal connections from 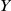 to 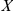 does not result in a natural causal effect and on the other hand side we can still compute the causal effect of 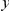 on 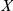 using the above formula. The key here is what we add by the modifier ‘natural’. To illustrate this, consider what would happen if we were to reconstruct the joint distribution of 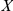 and 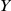 from the marginal distribution of 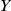 and the distribution of the interventions given in Eq. 7. That is, consider the joint distribution
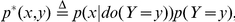



with the additional constraint that we choose the distribution of interventions according to Eq. 5. Now given the formula in Eq. 7 we see that 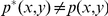 (unless, of course, if 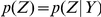, but this condition is not compatible with Figure 2B, that is, with 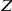 being a common driver). This means that even if we make sure that the marginal distribution of 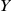 is the correct one, we cannot reconstruct the observed natural joint distribution and hence the natural ’dynamics’ of the variables in the system. In other words, the interventions change the system and the causal effect is with respect to this changed system. As mentioned above, sometimes this is indeed what is desired but in most cases where causality analysis is applied to the neurosciences the aim is to characterize what we have called the natural causal effect.

Apart from causal effects of the form 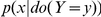, one could argue that for cases like the one in Figure 2B, it would be relevant to consider *conditional* causal effects 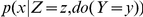. That is, given that 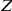 is a confounder, a way to get rid of its influence is to examine the causal effect for each observed value of 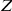 separately. In analogy with the definition of natural causal effect above we define the *conditional natural causal effect* of 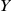 on 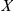 given 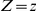 to be
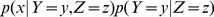
(8)


if and only if
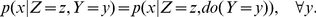
(9)


Eq. 9 is analogous to Eq. 2 and constitutes a criterion for the existence of the conditional natural causal effects. Furthermore, in Eq. 8 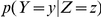 should be interpreted as 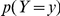 in Eq. 5, being the distribution of the interventions related to the criterion of maintenance of the natural joint distribution. It is important to note the different nature of this causal effect with respect to the unconditional one. In effect, for each value of the conditioned variable 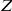 there is a (potentially) different natural causal effect of 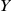 on 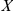. That is, in this case it does not make sense to talk about the causal effect of 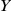 on 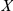, instead the causal effect is of 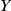 on 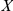 for 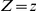. In contrast to the criterion of Eq. 2 this criterion (i.e. Eq. 9) is fulfilled in the causal graph of Figure 2B (see Supporting Information S1 for the details). We will further address the interpretation of unconditional and conditional natural causal effects in a subsequent section below. In Supporting Information S1 we show the conditions under which Eq. 9 holds. Like for the unconditional case one of the requirements is that there are no causal connections in the opposite direction (i.e. from 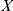 to 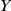). The other condition is analogous to the lack of common drivers in the unconditional case. In particular, the influence of any possible common driver should be blocked by the conditioning on the variables in 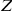, or in technical language, 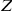 satisfies the back-door criterion relative to 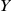 and 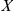 (see Definition 3.3.1 in [10]).

We have defined the natural causal effect from one (set of) variables to another as their joint distribution. In practice it will often be more convenient to characterize the natural causal effect with a lower-dimensional measure. Below we will indicate some possible such measures. However, note that the emphasis in this work is not so much in applying this framework to data but to show under which conditions the interactions between subsystems can be given a cause-and-effect interpretation.

### Natural causal effects between brain dynamics

Introducing natural causal effects above we considered 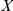 and 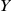 to be univariate or multivariate random variables. In the case of studying the interactions between different subsystems of the brain we are led to consider natural causal effects between time series. We assume that the variables in the time series are causal, that is, the variables in one time series can potentially have direct causal influence over variables in the other. Since we are modeling a system (e.g. the brain) without instantaneous causality, we will not include instantaneous causality in the models below. (Note that in applications it might be important to have a high enough sampling rate to avoid ‘instantaneous causality’.) Given two subsystems 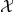 and 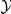 with time changing activities we let 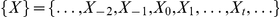 and 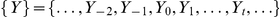 denote two time series corresponding to the activities of 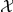 and 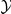. That is, relative to some temporal reference frame, 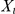 is the random variable that models the activity in 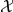 at time 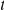.

When asking ‘causality questions’ about time series it is important to be specific about exactly what is the type of causal effect of interest. In particular, one could be interested in causal effects at different scales. For example, the interest could be in the causal effect of 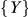 on 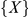, which would then be viewed as the impact of the totality of the causal connections from 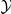 to 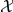. Indeed, this seem to be the causal effect that has received most interest in neuroscience (e.g. [8]). Alternatively the interest could be in the causal effect at a particular point in time, e.g. we could ask about the causal effect of 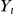 on 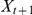. Of course, these two types of causal effects at different levels of descriptions are related, but are not equivalent and reflect different aspects of the impact of the causal connections between the subsystems.

We will use the framework of causal graphs to represent the causal structure of the dynamics of the subsystems. Since we assume that there is no instantaneous causality, 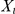 cannot interact directly with 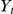 (and vice versa). We use
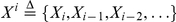



to denote the past of 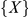, relative to time 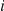. Furthermore, for simplicity we only represent direct causal connections of order 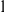 in the causal graph (i.e. from 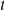 to 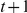), but our results are generic.

**Figure 3:**
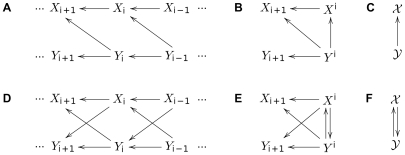
Graphical representation of causal connections for subsystems changing in time. Causal graphs represent two subsystems with unidirectional causal connections from 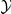 to 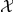 (A–C), or bidirectional causal connections (D–F). From left to right the scale of the graphs changes from a microscopic level, representing the dynamic, to a macroscopic one, in which each subsystem is represented by a single node.

The subsystems can be represented at different scales, according to the type of causal effect one is interested in. We will consider the case of two subsystems with unidirectional causal connections from 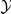 to 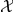 (Figure 3A–C), or alternatively with bidirectional causal connections (Figure 3D–F).

The *microscopic* representation of the causal structure displays explicitly all the variables and their causal connections (Figures 3A,D). At this microscopic level the graph is always a directed acyclic graph (DAG), given the above assumption of no instantaneous interactions. The microscopic level is required to examine types of causal effects that consider some particular intervals of the time series, e.g. 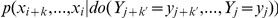. If instead we consider the time series 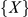 and 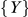 in their totality we get the *macroscopic* causal graph shown in Figure 3C,F. The macroscopic representation is useful because of its simplicity, with only one node per system, and it has been often used in the literature e.g. [20]–[22]. Note that at the macroscopic level a microscopic DAG can become cyclic (Figure 3F). At the macroscopic level the only causal effect to consider is 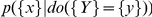. As one intermediate possibility we could consider a *mesoscopic* representation (Figure 3B,E). Here the ‘past’ of the two time series at some point in time (referred to by 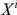 and 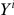, respectively) is only implicitly represented, whereas the ‘future’ (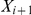 and 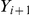) is explicit. The causal effects related to this representation are of the type 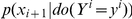, and the conditional causal effects 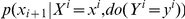. As we will see below, this is the representation that best accommodates Granger causality.

Different levels of representation may be used depending on the type of causal effects to be studied. However, it is important to emphasize that the conditions for the existence of natural causal effects give consistent results independently of the scale of the representation. For example, whether the natural causal effects 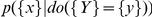 exist or not can be checked using the microscopic causal graph. This is because the representations at the different levels are consistent, so that an arrow in the macroscopic graph from 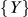 to 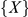 exists only if any directed causal connection from 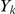 to 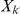 exists. This consistency reflects that at the macroscopic level the time series are conceived, not just as a set of random variables, but as representative of the dynamics of the subsystems. While at the microscopic level the status of the relation of a variable 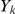 with another 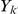 may seem equivalent to the one with 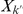, being this relation determined by the causal connections, the consideration of the time series as an entity breaks this equivalence, because the variables 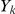 and 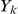 can be merged as part of the time series 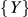, while 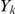 and 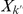 are not considered together at the macroscopic level.

At the macroscopic level, the natural causal effect from time series 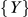 to 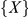 is, in analogy with Eq. 3, given by
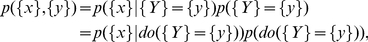
(10)


and can be seen as reflecting the total influence of the dynamics of the subsystem 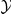 to the dynamics of 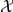. However, as we mentioned above, this high-dimensional causal effect is not the only type of causal effects that can result from the causal connections from 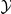 to 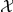. Other types of causal effects related to distributions of lower dimension, like the ones mentioned above, also reflect some aspects of the impact of the causal connections. This diversity of types of causal effects indicates that the causal structure should be seen as a medium that channels different types of natural causal effects. The idea of quantifying causal effects with a single measure of strength is an oversimplification, and although in some circumstances focusing on one of these types of natural causal effects may suffice to characterize the dynamics, in general they provide us complementary information.

The causal graphs at different scales can be related to different types of models. Macroscopic causal graphs have been used to represent structural equation models (SEM), where time is ignored. This type of models have been described in detail in the interventional framework of causality (see Chapter 5 in [10]), but there is no fundamental limitation of this framework to functional models that do not take time into account. In fact, sequential time interventions have also been studied (e. g. [23]) and recently the relation between interventions and Granger causality in time series was considered [24]. See also the so-called Dynamic structural causal modeling in [14]. Once time is explicitly represented like in the microscopic scale, the acyclic structure of the graph is not incompatible with the representation of feedback loops between the subsystems.

### The constraints of the causal structure on the existence and characterization of natural causal effects between dynamics

Here we will consider in more detail when natural causal effects exist and can be characterized, and thus when the question "What is the causal effect of the subsystem 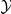 on the subsystem 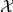
?" is meaningful. For simplicity we will restrict ourselves to the bivariate case illustrated in Figure 3.

Consider first the case of unidirectional causal connections from 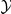 to 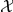 in Figure 3A–C. For any type of causal effect that involves an intervention of some variables of the time series 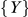, generally 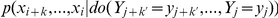, we need to examine if they are common drivers between 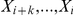 and 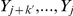. Due to the unidirectional causal connections all the common drivers are contained in 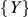. This means that any conditional causal effect 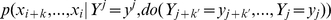 is a natural causal effect. Furthermore, the criterion of existence is also fulfilled for 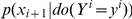 and 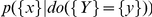 (See Supporting Information S1). In all these cases one can select the marginal distribution of interventions in agreement with the natural distribution according to Eq. 5 and thus preserve the natural joint distribution (Eq. 4). This implies that in the case of unidirectional causality it is possible to quantify the impact of the casual connections in terms of the causal effect of 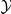 on 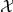. In the next section we will indicate how this can be done.

Consider now the case of bidirectional causal connections in Figure 3D–F. At the microscopic level we see that for 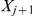 and each variable 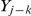, 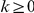, the variables 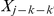, 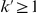 constitute common drivers (direct or indirect). This is also reflected in the mesoscopic time scale, where 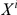 is a common driver of 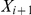 and 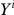 and there is a loop between 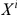 and 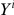. Therefore, the criterion of existence of the natural causal effects is not fulfilled in the case of bidirectional causality.

By contrast, the criterion of existence is fulfilled for the conditional natural causal effects, for example 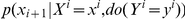 that we mentioned when we introduced the mesoscopic level. In this case all the possible common drivers, contained in 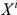, are conditioned. Therefore we can say that there are conditional natural causal effects from 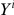 to 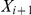 given 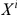 even in the case of bidirectional causality. However, to determine if this type of conditional causal effects can be used to characterize the impact of the causal connections from 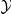 to 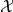, we still have to consider the preservation of the joint conditional distribution (Eq. 8). In analogy to Eq. 5, to preserve the natural dynamics we need to select the interventions according to
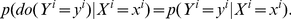
(11)


That is, we have to choose the interventions conditionally upon 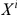, but this is clearly contradictory to the idea of defining a causal effect from 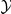 to 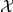. Since 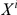 conditions the interventions, we cannot interpret the causal effect as representative of the impact of the causal connections from one subsystem to the other. This is because we do not simply consider the conditional effect of a set of variables 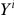 in a variable 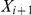 given a another set of variables 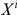. The variables 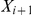 and 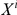 are related since we consider them as part of a single entity, namely the time series 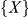. The conditional natural causal effects 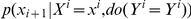 with a distribution according to Eq. 11 occur given how the causal connections generate the observed dynamics, but these conditional causal effects cannot be understood as being from one subsystem to the other. In particular, the distribution of the natural interventions is also determined by the causal connections from 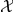 to 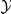.

Altogether, in the case of bidirectional causality, none of the candidate causal effects considered fulfills the criteria for the existence of natural causal effects and the maintenance of the joint distribution. For bidirectional causality the causal effects like 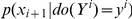 and 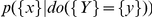 do not exist as natural causal effects, while conditional causal effects like 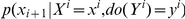 take place in the system, but cannot be understood as causal effects between the subsystems.

We emphasize that what prevents the interpretation of the conditional natural causal effects discussed above as a causal effect between the subsystems is to some extent the point-of-view of the interpreter. What our analysis show is that problems of interpretation arises when grouping variables together as when considering 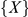 as standing for the activity of a particular subsystem. At the microscopic level, keeping time locality, one can analyze these conditional natural causal effects. For example, 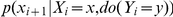 can be viewed the natural causal effect of 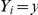 on 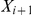 given 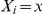. That is, relative to a particular time instant, we can meaningfully talk about conditional natural causal effects. However, note that since 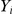 does not represent the dynamics of 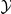, this natural causal effect cannot be considered the effect of 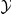 on 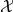.

That we cannot in general answer what is the causal effect of a brain subsystem on another while processing some stimulus or performing some neural computation may seem surprising. However, the definition of the natural causal effects (Eq. 3) was derived precisely to be consistent with what should be expected from a definition of causal effect to be used to analyze the natural activity of brain dynamics. We will now give some arguments to indicate that the restrictions imposed by the causal structure are in fact intuitive.

In Figure 3 we showed that, while at the microscopic scale a DAG is obtained as long as instantaneous interactions are excluded, at the mesoscopic and macroscopic scales the existence of bidirectional causality leads to a cyclic graph (Figure 3E–F). This means that 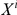 and 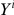 are mutually determined. This mutual determination can be understood as the impossibility to write a set of equations such that the dynamics of 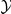 can be determined previously without simultaneously determining the dynamics of 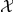. At the microscopic scale, for bidirectional causality (Figure 3D), one can consider a path from some node 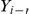, 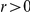, to 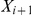 which is directed and thus follows the causal flow, but contains both arrows 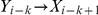 and 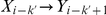. In this case it is not possible to disentangle in the natural dynamics the causal effect in the opposite directions: when considering the causal effect from 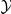 to 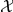, assuming 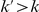, it is not clear to which degree the influence of 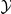 on 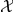 is intrinsic to 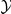 or due to the previous influence of 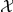 on 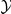.

That is, in the case of a bidirectional coupling, 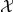 and 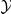 form a unique bivariate system in which the contribution of one system to the dynamics of the other cannot be meaningfully quantified. Therefore it does not make sense to ask for the causal effect from one subsystem to the other, but to examine how the causal connections participate in the generation of the joint dynamics. A simple neural example illustrating this view would be the processing of a visual stimulus in the primary visual cortex (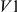). For example, responses of 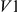 cells are influenced by the spatial context of the the stimulus due to the feedback projection from cortical area MT (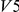), where receptive fields are much larger than in 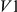. This means that there is a feedback modulation from a higher level in the visual pathway, which activity depends itself on the processing in 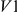 [25]. In this case what is important is to understand how the causal structure, and in particular the existence of the feedback causal connections, are necessary to generate the regime of the responses and to obtain some characteristics of the neurons' responses that are functionally relevant, like the influence of stimulus context.

### The analysis of causal effects

We have complemented the definition of causal effects from the interventional framework with two criteria that reflect the requirements specific for studying causality between subsystems of the brain during their natural activity. In this section we consider how to quantify the natural causal effects between dynamics and more generally any causal effect related to a change in the dynamics. The measures introduced below should be considered devices for further analysis and characterization of natural causal effects and not necessarily as tools for data analysis.

We start by considering how to quantify single causal effects of a given type, for example 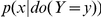 for a fixed intervention 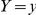. Assume that we check in a causal structure that this type of natural causal effects exists. To quantify this causal effect we need a reference distribution to which the post-intervention distribution can be compared. The appropriate reference distribution will typically be dictated by the context. For example, in Eq. 1 the reference was the marginal distribution 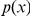, which is just the average over all interventions. Alternatively, one can consider the same intervention 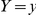 but for a different configuration of the system, in which some causal mechanisms are assumed to have changed. In this latter case we denote the reference distribution 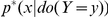. Notice that even when a type of natural causal effects exists and thus the impact of the causal connection from 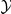 to 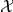 can be interpreted in terms of cause-and-effects, one typically cannot quantify the causal effects in absolute terms. Only in some special cases, when the reference distribution can be associated with the absence of causal interactions, can the causal effect be interpreted in absolute terms. In this case, and only in this case, can we talk of the *strength* of the causal effect. In the general case, the measures we consider here reflect relative differences of the causal effects.

Our analysis of natural causal effects relies on the comparison of probability distributions but we emphasize that the first step in the analysis must be to make sure that the type of natural causal effects studied actually exists in the system. To compare two probability distributions associated with natural causal effects one can use different measures, as for example the expected values as in Eq. 1. Here we will use Kullback-Leibler divergences (see Methods) since these are sensitive to all the moments of the distributions and also because this allows for a more direct comparison with a general measure of Granger causality, the transfer entropy (see below). For example, in the case that the same intervention for another configuration is used as a reference we can use:
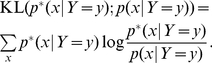
(12)


Here we have assumed that the domain of 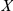 is the same in the two configurations. When this is not the case, Jensen-Shannon Divergence should be used instead (see Methods). The Kullback-Leibler divergence 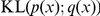 is an asymmetric measure that quantifies how different 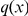 is from 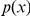. If 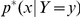 corresponds to the reference distribution associated with no causal interactions, the particular form of Eq. 12 enables an absolute comparison of natural casual effects for different configurations. Note, however, that we are not interested in characterizing a particular measure but in examining the principles of the analysis of natural causal effects. The particular measure selected for application to data should also depend on other issues such as the balance between the degree of sensitivity to differences between the distributions (i. e. linear vs. nonlinear measures), and the difficulty to estimate the measures from limited amount of data.

We now consider how to quantify the natural causal effects in a system, when different causal effects of the same type have to be taken into account. As discussed previously, the impact of causal connections does not depend on single interventions, but on all the different natural causal effects of the same type occurring in the dynamics with a given probability distribution. How to proceed to compare these different natural causal effects depends on the purpose of the analysis. For example, one may be interested in quantifying the average difference of the natural causal effects for two different configurations. In this case one can use the probability of the natural intervention (Eq. 5) to average Eq. 12. That is, one could use
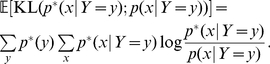
(13)


This analysis can be done indistinctively of which type of natural causal effects is analyzed. For processes one can substitute 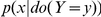 by for example 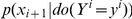 in Eqs. 12 and 13. Again the requirement for these measures to be interpreted as quantifying the natural causal effects between the processes is that the criteria discussed above are fulfilled.

### How Dynamic Causal Modeling and Granger causality answer the questions about causality

We will now review how the three different questions about causality introduced above are addressed by DCM and Granger causality, the two main approaches that are commonly applied to study causality between the brain systems. Although this section is not strictly a ‘Results’ section, casting DCM and Granger Causality in terms of the framework we propose will make it possible to see (in the next section) how our work extends and complements these approaches. Before examining DCM and Granger causality separately, we should note that both have in common that they rely on the statistical analysis of observational data. Both are thus ultimately limited by the existence of observationally equivalent causal structures with regard to the inference of the causal connections (question Q1 about existence). Here we will not consider these limitations further but instead focus on how DCM and Granger causality address question Q3 about the quantification of causal effects. We will focus on the essential aspects of these approaches as formulated by [26] and [27], respectively.

Dynamic Causal Modeling is based on modeling the observed data, and from our perspective it can be considered an approach to answering Q2. Since DCM explicitly considers the dynamics, in contrast to previous modeling approaches such as SEM, it represents an important step towards obtaining a model that can faithfully reflect the causal structure and the dynamics of the real system. Similarly, since it considers a forward model that maps hidden states to observed quantities it also is an improvement with respect to the approaches based on the parametric autoregressive model formulation of Granger causality [28]. In DCMs the state equations are causal in the sense that the rate of change of the variables generally depends on the state of the system. This means that a causal structure at the macroscopic scale can be constructed from the state equations. The form of the state equations in DCM makes an interventional interpretation of the model possible due to the asymmetry between the left- and right-hand terms in the equations [14]. Nonetheless, the correspondence of the inherent causal relationships in the model to the causal structure of the real system depends critically on how accurate the state equations model the underlying neuronal activity. With regard to Q1 it remains as an open question how realistic a model should be so that the causal structure of the system can be inferred correctly. Furthermore, in practice, since typically only few subsystems are modeled, latent variables are certainly present and could introduce “spurious causality”, for example making two subsystem appear causally related whereas in fact they are not (see [29] and [14] for discussion of the missing region problem in the concrete context of fMRI analysis). This is the ultimate limitation when inferring causality without intervening mentioned above.

We are here mainly interested in how DCM addresses question Q3 about the causal effects. Effective connectivity is generally understood as the influence one region exerts over another [8]. In practice though, this abstract definition is made concrete considering that the effective connectivity is associated with the coupling parameters between variables representing different brain regions in the state equations [26]. For example, for two different conditions, a model is fitted and the gain of the coupling parameter that models the connectivity from region 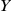 to region 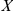 is reported. Therefore, the *strength* of the causal connection is analyzed in terms of the coupling parameters in the model. There is no explicit definition of the causal effect. The change in the coupling parameter is discussed in terms of adaptation or stimulus modulation of the connectivity, thus focusing on the mechanistic change, without considering its impact in the dynamics. In some simple cases, such as when the state dynamics are linear, one can infer interventional causal effects from the model parameters [14]. However, it is important to emphasize that a functional (or biophysical) model (dynamic or not) of the real system does not alleviate the restrictions with respect to natural causal effect imposed by the causal structure. In other words, if a particular causal structure is incompatible with a natural causal effect from 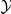 to 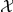 the values of parameters describing the coupling from 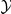 to 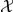 can not be interpreted as reflecting the causal effect of 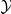 on 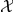 in the natural dynamics, even if they are informative about what would be the causal effect of an external intervention of 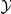 on 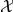.

The approach of Granger causality is significantly different from the one of DCM. Many years ago Sir Clive Granger suggested a criterion for testing for causality between time series [28]. In the bivariate case, this criterion says that there are no causal connections from process 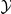 to process 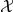 if and only if
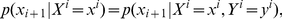
(14)


where 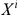, 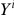 refer to the whole past of 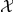 and 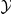, respectively. In words, 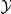 is causing 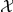 if the future of 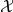 given the past of 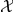 is not independent of the past of 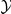. Therefore the criterion of Granger causality tests for a conditional independence, which corresponds to the type of constraints we mentioned above that the causal structure imposes on the statistical dependencies. Given that these constraints are generally not enough to infer the causal structure the criterion of Granger causality is only applicable under some assumptions. We will called these assumptions *complete observability*. What is assumed is that there is no hidden process which is a common driver of 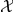 and 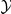. This assumption is related to the fundamental limitations of causality inference from observational data and is common to DCM. Furthermore it is assumed that we have access to the relevant processes between which the causal connections exist, so that the probability distributions are estimated for variables directly corresponding to these processes. This assumption avoids the use of a forward model that maps hidden states to observed quantities. This second assumption is specific of Granger causality in contrast to DCM where such a forward model is explicitly included.

In the original formulation, Granger used the mean of the distributions to test the above equality, but it was clear to him that other measures could be used as well [27]. The most general test of the equality of the two probability distributions in Equation 14 uses the Kullback-Leibler divergence (See Methods). The resulting measure has been introduced several times in the past and in different contexts [30]–[32]. Most recently, the same measure was re-introduced under the name ‘transfer entropy’ [33] and due to its recent popularity, we will use this name in the sequel. See e.g. [14], [34] for a more detailed description of how different formulations of Granger causality appeared in different fields and for different types of processes. In particular we can formulate Granger's causality criterion (Eq. 14) as a comparison between 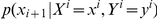 and 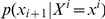 using the KL divergence. The transfer entropy from 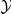 to 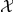 is:
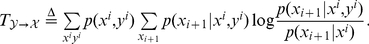
(15)


Note that due to a basic property of the KL-divergence the transfer entropy is zero if and only if the criterion of Eq. 14 is fulfilled.

We now consider how Granger causality analysis addresses the different questions about causality. Since it provides a criterion to infer the existence of the causal connections it focuses on answering Q1. However, notice that the Granger causality criterion is not designed to infer a causal graph that considers explicitly the temporal dynamics (the microscopic scale in Figure 3). The Granger causality criterion only intends to infer if there is any causal connection from 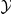 to 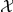, that is, allows us to construct the macroscopic causal graph.

Granger causality is based on a criterion for causal inference and thus in its more general nonparametric formulation does not involve modeling, so that question Q2 is not addressed. The transfer entropy constitutes the most general measure to test for the criterion of Granger causality. This means that theoretically a nonzero 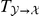 implies the existence of some causal connection from 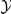 to 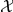, thus answering Q1 (if the assumption of observability are fulfilled). In practice, one needs a way to assess the significance of the nonzero value and in general bootstrapping or surrogates are needed (e. g. [5], [35], [36]). If instead of transfer entropy the parametric formulation using linear autoregressive models is applied, one could consider that question Q2 is also addressed but this means assuming that the autoregressive model is realistic enough to reflect the causal mechanisms.

Regarding Q3, the same statistic used to test for causality is commonly used to quantify the strength of the causal connections. For example, Granger (1963) [37] refers to the Granger causality measure for linear Gaussian processes as the *strength of the causality* from 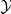 to 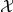. This idea of strength suggests that, apart from assessing the significance of a nonzero value, one should use the value of the statistic for quantification. As for DCM, there is no explicit definition of what the causal effects are. However, in Granger causality, the causal effect is quantified taking into account the dynamics of the processes, instead of examining the changes of single coupling parameters. The emphasis is put on the impact of the causal connections on the dynamics of the processes, so that the causal effects are implicitly conceived as a result of how the causal connections participate in the generation of such dynamics.

Our definition of natural causal effects between processes is closer to this implicit notion of causal effects used in Granger causality, since it also considers the impact of the causal connections on the dynamics, in contrast to DCM that compares coupling parameters of the model. However, we also argued that this impact cannot be captured with a single measure of strength. Oppositely, the causal structure results in different types of natural causal effects going on in the dynamics, which are associated with different aspects of how the dynamics arise from the causal structure.

Furthermore, the constraints for the existence of natural causal effects, that for example determine that natural causal effects between processes do not occur in bidirectionally coupled bivariate systems, are contradictory with the common practice of comparing the causal effect in both directions e.g. [6], [38]–[42] when applying the Granger causality analysis. It can be shown that this contradiction is due to a misuse of Granger causality measures and in particular of transfer entropy as measures of causal strength [43]. A detailed description of why transfer entropy cannot be generally used for the quantification of causal effects and under which conditions it has a meaningful interpretation in terms of natural causal effects is left for a future contribution. We will next discuss to which degree the analysis of causal effects we propose should be considered as complementary or substitutive of DCM and Granger causality.

### How to combine the analysis of natural causal effects with Dynamic Causal Modeling and Granger causality

The approach we proposed for the quantification of natural causal effects should be used instead of Granger causality measures to address the question of quantification (Q3). This means that the transfer entropy should be only used as a statistic to test the existence of the causal interactions based on the general criterion of Granger causality (Eq. 14). Even for inference one should be aware of the strong limitations imposed by the assumption of complete observability. In the interventional framework the limitations of Granger causality and thus transfer entropy to infer the causal structure without complete observability are well known. Alternative measures of information flow have been proposed [17] and compared to transfer entropy [43].

Although the value of the transfer entropy cannot be used as a measure of strength of the causal effects under some conditions that depend on the causal structure it quantifies the information transfer from one process to another [43], while more generally it can still be used to characterize the temporal statistical dependencies in the signals. Bressler and Seth (2010) [44] distinguished between *effective connectivity* [8] and a different concept of *causal connectivity*. While the *effective connectivity* is expected to reflect the causal influence one brain area exerts on another, *causal connectivity* is considered more pragmatically as a description of directed dynamical interdependencies present in the recorded signals. Although using the term *causal* can be misleading in this context, the transfer entropy, considered strictly as a statistical measure, has a rigorous meaning in terms of information loss [45] and can capture aspects of the dynamics which may be less reflected in symmetric measures (like coherence or symmetric mutual information). Therefore, the type of analysis related to Eqs. 12 and 13 should substitute Granger causality to analyze causal effects in the natural dynamics, but it is complementary to the use of transfer entropy as a measure of information loss.

Our approach is also complementary to Dynamic Causal Modeling: it can be used to examine the dynamic impact of the changes across conditions of the coupling parameters associated with effective connectivity. In particular, the only thing specific for the analysis of natural causal effects in Eq. 12 is the selection of probability distributions that are associated with natural causal effects. Alternatively, one can relate the two compared configurations to a change in a coupling parameter. The comparison of a particular probability distribution across configurations (not necessarily associated with natural causal effects) shows the impact of this change on a particular aspect of the dynamics. For example, one can compare 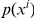 when a coupling parameter associated with the strength of the causal connection from 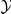 to 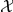 changes:
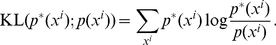
(16)


This change of a coupling parameter can be seen exactly as a punctual intervention. Although in causal graphs associated with the state equations of a DCM model, the coupling parameters do not appear as nodes, if two alternative models are compared they can be merged in a single model where the parameter can be seen as a binary variable, so that choosing one or the other model is equivalent to intervening this variable to one of the values. Therefore the comparison of a particular type of probability distributions like in Eq. 16 quantifies the causal effect of the change in the coupling parameter. This type of causal effect is not a natural causal effect from one brain subsystem to another that occur as part of the generation of the dynamics; it is the causal effect that a change in the mechanisms has on some aspects of the dynamics related to the probability distributions chosen. Also here there is flexibility to examine different aspects of the dynamics selecting different probability distributions, for example 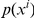, or 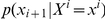, in the same way that the natural causal effects from 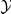 to 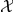 are studied examining different distributions like 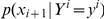 or 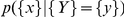. This type of analysis complements the direct comparison of the coupling parameters (effective connectivity) because it is not obvious without actually examining the distributions to derive how a change in the parameters affects the dynamics.

DCM addresses question Q2 about the mechanisms aiming to provide a realistic model of how the dynamics are generated. In this regard, the analysis of the causal effects considered above cannot substitute the DCM approach. However, considering that only in some cases the natural causal effects between brain regions exist helps to bound the meaning of effective connectivity, generally understood as the influence one region exert on another. The coupling parameters can be related to causal effects of external interventions [14] but do not quantify natural causal effects occurring in the recorded dynamics. Furthermore, the analysis we suggested above can straightforwardly be extended to examine the causal effect of a change in a coupling parameter on some aspects of the dynamics, something which is not easy to evaluate from the comparison of the coupling parameters across conditions. We will illustrate this point further when analyzing causal effects in an example system below.

### Testing for causality and analyzing causal effects in a simple model

We now examine a model system to illustrate the distinction between the inference of causality and the analysis of the causal effects. Here we focus on a simple example of a stationary Markov binary process. In Supporting Information S1 we study the case of linear Gaussian stationary stochastic processes. We note that in these examples all the measures used are calculated analytically (see Methods) to isolate the fundamental properties of the measures from issues related to estimation from data. We consider the transfer entropy 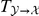 and two measures related to Eqs. 13 and 16, respectively. In particular, instead of the Kullback-Leibler divergence used in these equations we calculate the Jensen-Shannon Divergence (JSD) (see Methods), since it is well defined for probabilities with different domains, as the ones resulting from the Markov process explained below.

So consider a stationary bivariate Markov binary process of order 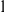. Both 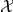 and 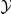 take only values 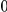 and 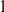. The process is completely determined by the transition probabilities and by the condition of stationarity:
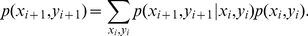
(17)


Furthermore, we assume that only unidirectional causal connections from 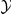 to 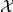 exist. Accordingly, the transition probabilities can be separated as the product 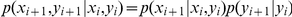. In particular, we let the transition probabilities for 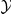 be 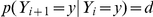, that is, 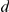 is the probability that the same value is taken at subsequent steps. The transition probabilities for 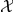 are such that 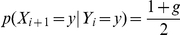, independently of the value of 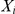. Therefore 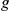 determines the strength of the connection from 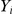 to 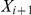, and there is a causal connection from 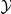 to 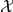 for 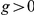. For 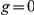, 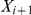 takes value 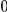 or 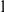 with equal probability and independently of 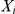 and 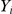. In the case 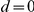, when 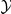 deterministically alternates between 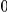 and 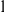, this example corresponds to one already discussed in Kaiser and Schreiber (2002) [46]. Here we present results for nonzero values of 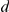 with different degree of stochasticity. We calculate the measures using 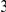 time lags for the past 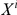 and 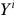, since this is enough for convergence and for higher lags the values obtained do not differ significantly. In fact, given that causal connections are only of order 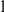, one time lag is enough when conditioning on 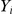. However, since in the transfer entropy (Eq. 15) the conditional entropy 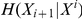 appears, where there is no conditioning on 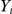, one has to consider all the information about 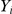 that exists in the past 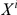.

**Figure 4:**
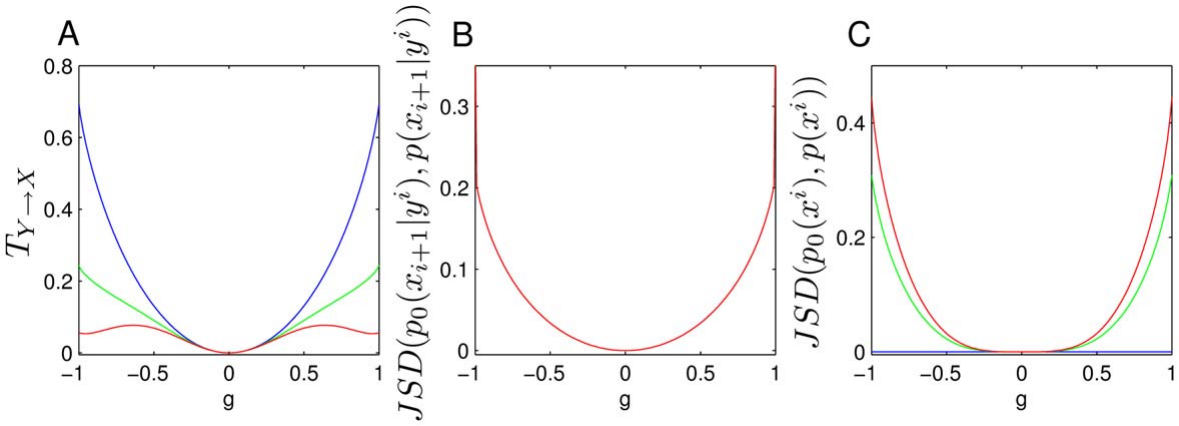
Causality analysis in a binary Markov process. Information theoretic measures used for the inference of causality and the analysis of causal effects calculated for a bivariate binary stationary Markov process of order 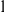. See the text for a description of the process. The measures are calculated analytically using 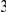 time lags to account for the past 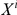 and 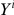. The results are shown for 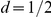 (blue), 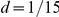 (green), and 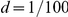 (red), where 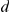 is the probability that 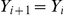. A: Transfer entropy 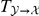 (Eq. 15). B: Jensen-Shannon divergence 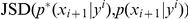 (Eq. 21). C: Jensen-Shannon divergence 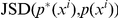.

First we examine how the transfer entropy 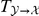 depends on 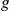 and 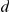 (Figure 4A). Supporting its use for the inference of causality from 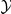 to 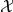, the transfer entropy 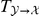 is zero if and only if 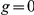. In the opposite direction 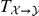 is always zero for this example (results not shown). In the Granger causality approach the transfer entropy is used also as a measure of the strength of the causal connection. From the Figure it is clear that the relation between the coupling parameter 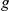 and 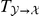 depends strongly on 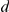. In fact, for low values of 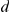, 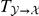 is nonmonotonic with 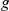. In words, the Granger causality measure is nonmonotonic with the parameter that determines the strength of the connection. This can be understood taking into account that transfer entropy quantifies the extra reduction of uncertainty that results from considering the past of 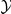 after considering the past of 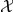. For low 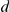 the dynamics of 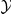 are almost deterministic, and thus when 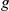 increases the dynamics of 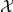 become also more and more deterministic. For such, almost deterministic, dynamics the remaining uncertainty of 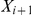 after conditioning on 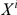 is already very small, and thus the extra reduction given 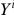 decreases with high 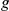. In fact, for the extreme values 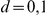 (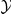 completely deterministic), the nonmonotonicity leads to 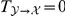 for 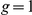 -see Figure 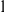 in Kaiser and Schreiber (2002) [46]. In such extreme cases transfer entropy cannot be used even to infer causal interactions. In fact, this limitation of transfer entropy in the inference of causality for strongly synchronized systems is well known e.g. [17], [47], [48].

In Figure 4B we show the Jensen-Shannon Divergence (JSD) for distributions of the type 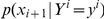. Since there is only unidirectional causality from 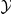 to 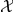 this distribution fulfills the criterion of existence for natural causal effects (Eq. 2), and thus the JSD can be used to quantify the changes in the natural causal effects from 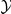 to 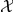 when 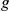 changes. This corresponds to the type of analysis described in relation to Eq. 13. Here the different configurations are identified by the value of 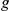. In particular we take the distribution 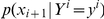 obtained for 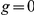, for which there is no causal connection, as a reference to compare the natural causal effects to. Notice that for this Markov process, since by construction there is no causal connection from 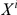 to 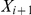, we have that 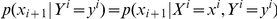. This means that the natural causal effects 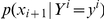 correspond to the distribution appearing in the numerator of the logarithm in the definition of the transfer entropy (Eq. 15). What is different with respect to the transfer entropy is the probability distribution used as a reference for comparison. Now the natural causal effects are compared across configurations. We see that the changes in the natural causal effect 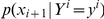 monotonically increase with 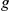 and are independent of 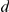. The independence of 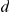 results from the particular generation of the process, since 
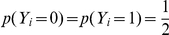
 independently of 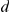 and since the causal interactions are of order 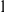 we have that 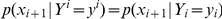. The monotonic divergence with respect to the distribution obtained for 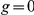 demonstrating that in this case there is a monotonic relation between 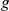 (the strength of the connection), and the impact of the causal connection (the natural causal effects). This should be contrasted to the results using transfer entropy described above.

In Figure 4C we show the Jensen-Shannon divergences for distributions of the type 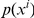 (Eq. 16). In contrast to the probability distributions 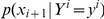, these distributions do not represent a natural causal effect that occurs in the dynamics. However, since the change of 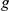 can be seen in itself as an external intervention of the system, we can compare 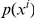 in dependence on 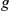 as a way to quantify the causal effect of this change in the model. As before we take as a reference the distribution obtained for 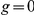. For 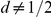, 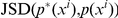 increases monotonically with 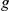 indicating that an increase in the strength of the connection renders the distributions more different. However, for 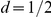 a constant zero value is obtained. Importantly, this should not be seen as a limitation of 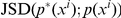 to quantify the causal effect, on the contrary it indicates that, with respect to the distribution 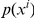, the changes in the effective connectivity (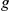) have no effect for 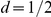. This illustrates how focusing on the value of a coupling parameter may be insufficient in order to describe the impact that a change has in a particular aspect of the dynamics.

## Discussion

In this work we have analyzed the applicability of the cause-and-effect framework to the study of natural dynamics of systems consisting of interacting subsystems. Our main result is that it is generally not possible to characterize the effects of the interactions for each subsystem separately. That is, the effect of causal interactions can typically not be described in terms of the effect of one subsystem over another. Rather, the interactions unifies the subsystems and creates a dynamics that transcends the limits posed by the individual systems. This result is generic in the sense that it only depends on the causal structure (i.e. on the topology of the causal connections) and not on the details of the system under study. Our work suggests that analyzing the effect of interactions in the natural dynamics in terms of cause-and-effect is of limited use, in particular in systems where the functional units tend to be heavily interconnected, such as the brain. We emphasize that our contribution should not be seen as a new method to substitute other approaches to causal analysis. The conditions of existence of natural causal effects indicate that inference of causal connections and analysis of causal influences should be considered different types of analysis with different requirements. When natural causal effects do not exist, they can not be quantified, no matter what measure is used. Our analysis therefore has important implications for all approaches aiming at characterizing the effects of causal interactions in the unperturbed system. We will now discuss our work in more detail and relate it to some previous work in the literature.

It might be helpful to first interpret our main result in the light of the three questions about causality introduced above. First, the existence of a causal connection from 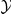 to 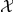 can always be probed. This is possible even if there are causal connections also from 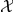 to 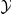. The ‘traditional’ way to do this is through interventions, that is by actively perturbing the 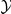 system. We pointed out that, under certain assumptions, Granger causality and DCM can be used to infer existence of causal connections from observational data. Second, the mechanisms by which these causal connections are instantiated can always be probed (at least in principle) by modeling the systems at the appropriate level. We note that interventions could play an important role also in this case. Indeed to constrain and corroborate the models, interventions might be very useful. Third, and this is our main result, the effect of the interactions naturally happening in the system can typically not be described as the effect of 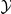 over 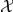 (or vice versa). Here, and this is a central point, interventions cannot be used (not even in principle) to quantify the effect of 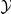 on 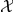 in the natural dynamics, unless they quantify a natural causal effect. Moreover, in this case our work shows the importance of using interventions that mimic the natural dynamics as closely as possible. Our results demonstrate that the quantification of causal interactions is a question separate from the other two questions and that it might not always be well defined.

Central to our results is the notion of a *natural causal effect*. This should be considered an adaptation of the interventional framework of causality [10] to the context of dynamically interacting subsystems. Whereas most work in the interventional framework focuses on causal effects resulting from external interventions, to study the unperturbed system we need a notion of causal effects from one subsystem to another related to the natural dynamics. A natural causal effect can be seen as a result of a naturally occurring intervention. In general, in the natural dynamics, different natural interventions of the same type occur with different probability. This led us to define the natural causal effect between variables as the observed joint distribution, and to consider a distribution of natural interventions determined by the marginal distribution of the intervened variable. Furthermore, we pointed out that there is no unique type of natural causal effect that can be used to study the impact of the causal connections between subsystems. Rather the causal structure constitutes a medium in which multiple types of natural causal effects arise.

The existence of natural causal effects and the maintenance of the natural joint distribution determine when the question about what is the causal effect from one subsystem to another in the natural dynamics is well-defined. This is in contrast to the question about the causal effect of an external intervention of one subsystem on another, which is not constrained by these criteria. We examined when these criteria are fulfilled in the case of two subsystems. We showed that natural causal effects from 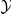 to 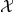 are well-defined if unidirectional causal connections from 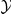 to 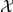 exist, but not in the case of bidirectional causality. Furthermore, our results indicate that some types of conditional natural causal effects exist even if the impact of the causal connections between the subsystems 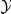 and 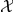 cannot be mutually disentangled. This is because they are defined based on an explicit consideration of time locality and are not compatible with the view of subsystems as macroscopic entities. In general the fulfillment of the criterion of existence depends on the causal structure (see Supporting Information S1). This means that only under quite restrictive conditions the natural causal effects exist and can be used to characterize the impact of the causal connections from one to another subsystem in the natural dynamics. A detailed analysis of multivariate systems remains for a future contribution but it is clear that the existence of natural causal effects will be even more limited in the case of more than two interacting systems. Notice that this limitation is of a fundamental nature, since it refers to the existence of the natural causal effects in the natural dynamics. This is in contrast to other limitations which are more of a practical nature, like the existence of hidden states for the inference causality [10], or computational time for model comparison [49].

We considered how the most used approaches to study causality in the brain address the three questions about causality and how compatible they are with the concept of natural causal effects. Granger causality should be seen as a criterion to infer the existence of causal connections between processes that is valid under the quite strong conditions of complete observability. However, Granger causality measures, including transfer entropy, cannot be used in general to quantify causal effects. The existence of a particular type of natural causal effects depends on the causal structure, and only when the causal effect exists a measure of its strength can be meaningful. Even when a type of natural causal effects exists and can be used for the characterization of the impact of the causal connection from 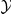 to 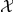 it only makes sense to consider the strength of the causal effects when one can compare to a configuration that corresponds to the case of no causality from 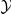 to 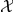.

We also introduced the idea of analyzing natural causal effects by comparing them across different configurations, i.e. different regimes of the same system (model). This approach was illustrated in a simple example system where the measures could be calculated analytically. Importantly, this type of comparison is not only useful to compare natural causal effects. When the different configurations are related to a change in the causal mechanism, comparing a particular type of probability distribution allows us to examine the impact of this change on the aspect of the dynamics captured by the probability distribution. Accordingly, the analysis of causal effects, which necessarily requires considering the dynamics of the system, complements the usual way in which Dynamic Causal Modeling is used to examine changes in the coupling parameters. The impact of these changes for some aspect of the dynamics is not easy to predict just from the form of the model, as was shown in the example.

The distinction between on the one hand the inference of causal connections and the modeling of causal mechanisms and on the other hand the analysis of causal effects is tightly related to the distinction between model fitting and model analysis. For example, in DCM it is common practice to fit a model for different configurations (that can correspond to different experimental settings related to different tasks) and then examine the gain in some coupling parameters associated with effective connectivity. Although this structural comparison is a first step for model comparison, this comparison should also involve comparing the dynamics that result from them. When meaningful given the causal structure, one can analyze the natural causal effects from one subsystem to another. More generally, even when it is not possible to disentangle the impact of the causal connections from 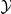 to 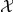 from the ones in the opposite direction and thus the subsystems are not separable, still the causal connections determine the generation of the joint dynamics. This means that in general the analysis of the impact of the causal connections cannot be formulated only in terms of causal effects from one part of the system on another, but examining the emergence of some properties of the dynamics. For example, it is well known that causal connections between two intrinsically non-oscillatory units can make them oscillate synchronously (e.g. [50]). That is, increasing the coupling leads to a qualitative change in the dynamics.

A conclusion from our work is that the notion of causal effects between subsystems might not be very useful in neuroscience. Given the ubiquitous existence of feedback and recurrent connections, the criterion for the existence of natural causal effects can hardly be fulfilled when analyzing neural data. Furthermore, it has been widely studied that these connections play important roles determining properties of the dynamics which are functionally relevant e.g. [25], [51]. Therefore, the impact of the causal connections in the brain is generally not related to causal effects from one subsystem to another. Even when considering the effect of an external sensory stimulus, which is closer to an external intervention, one is not interested on the impact of this intervention *per se*, but in which way the brain is capable of encoding and decoding the sensory information. This perspective follows the idea suggested by [52] that, regarding the neural code and neural computations, statistical dependencies are more relevant than causal effects. In the same line [43] illustrated that while a measure of causality based on interventions [17] can be informative about the causal structure when the transfer entropy provides erroneous information about it, the transfer entropy, as a measure of statistical dependence quantifying the extra reduction in uncertainty when considering the past of the other process, is in general more informative about the computational properties of the system. This means that in fact, when the transfer entropy is used to study causal interactions between brain regions from experimental data e.g. [6], [38]–[42], or neural models e.g. [53]–[55], it may be not only more correct but also more useful to interpret this measure not in terms of the strength of causality but as a measure of statistical dependence. Furthermore the transfer entropy is connected to the mutual information rate [56], [57] and under some conditions it quantifies information transfer [43].

Pearl's interventional approach to causality constitutes a unifying framework that relates different approaches to causal analysis like counterfactual analysis [9] and structural equation modeling [58]. However, the consideration of sequential interventions [23] or time series is less common in the interventional framework. Only recently the link between Granger causality and the effect of interventions has been examined [24] in detail. Also for Dynamic Causal Modeling the interpretation of the coefficients in a bilinear model in terms of single external interventions has recently been pointed out [14]. Examining the way in which this general approach is compatible with the aims of studying causal effects between brain subsystems helped us to clarify when it is meaningful to ask for the causal effect from one brain subsystem to another, and to distinguish what type of information this analysis provide us in comparison to the inference of causal connections or the analysis of dynamic statistical dependencies between the subsystems.

What is new in our approach with respect to the interventional framework is the idea of considering when causal effects occur naturally in a system. This also led us to consider probability distributions of interventions, while Pearl focuses on the analysis of single interventions [10]. The novelty of our contribution, based on the proposal of the criteria of the existence of natural causal effects and the maintenance of the natural joint distribution, results from the different aims that the analysis of causal effects between brain regions has with respect to other applications envisaged in the development of interventional causality. In general, in medical research e.g. [59] or epidemiology [60], one is really interested in assessing the effect of external interventions that alter the system. However this is not in general the case when studying causality in the brain, at least for the type of analysis in which Granger causality or Dynamic Causal Modeling are commonly applied.

We expect this work to contribute to clarify the aims and potentials of causal analysis applied to study brain dynamics and to complement a recent vivid debate about brain causality [14]. Understanding the links between causal structure, causal effects and statistical dependencies is a line of research complementary to the development of more accurate models of brain dynamics [61]. We have written this paper in the context of neuroscience but the concept of natural causal effects should clearly be useful in other fields where effects of interacting subsystems are of importance.

## Methods

We used tools from information theory (e.g. [45]) to characterize the similarity of two probability distributions and the statistical dependencies between variables. The basic quantity is the Kullback-Leibler divergence (KL divergence). The KL divergence is a measure of the difference between two probability distributions and is defined as
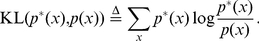
(18)


An important characteristic of the KL divergence is that it is a non-negative number and is zero if and only if the two distributions are identical. The mutual information
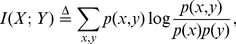
(19)


is a particular average of KL divergences
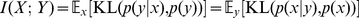
(20)


that quantifies the interdependence between the random variables.

We have considered two examples, one in the main text and one in Supporting Information S1. In these examples we calculated KL divergences for some particular distributions resulting from stationary processes for which these measures can be calculated analytically. For the bivariate Markov binary stationary processes we used the Jensen-Shannon divergence (JSD) instead of the KL divergence because it is well defined for probabilities with different domains [62]. The JSD corresponds to an average of two KL divergences:
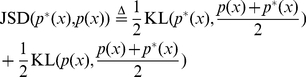
(21)


and is bounded between 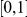.

For the bivariate linear Gaussian stationary processes the KL divergence can be expressed in terms of the mean and covariance matrix of the distributions. This can be derived in analogy to the entropy of a multivariate Gaussian distribution (see Theorem 8.4.1 in [45]). For two Gaussian distributions 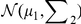 and 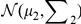 The KL divergence is:
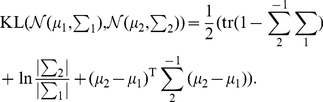
(22)


## Supporting Information

Supporting Information S1 Supporting text.
(PDF)
