# Supplementary material for: When Two Become One: The Limits of Causality Analysis of Brain Dynamics
Source: PLoS One. 2012 Mar 16;7(3):e32466. doi: 10.1371/journal.pone.0032466 (PMC3306364; doi:10.1371/journal.pone.0032466)
Supplement: Supporting Information S1 — Supporting text. (PDF) [file pone.0032466.s001.pdf]

Supporting Information for When two become one: Limits of causality analysis of brain dynamics

Daniel Chicharro[1*], Anders Ledberg[1*]
1 Center for Brain and Cognition, Department of Information and Communication Technologies, Universitat Pompeu Fabra, Barcelona, Spain
∗ E-mail: chicharro31@yahoo.es, anders.ledberg@gmail.com,

Contents

S1 Calculation of causal effects from observed probability distributions **2**

S2 Exemplary calculations of causal effects **3**

S3 Conditions for the existence of natural causal effects **5**

S4 Analysis of causal effects in exemplary linear Gaussian stationary processes **7**

List of Figures

S1 Causal graphs illustrating the effect of interventions 3
S2 Causality analysis in an AR(1) model. 8

S1 Calculation of causal effects from observed probability distributions

We here show how to express a postinterventional probability in terms of the observed probabilities when it is assumed that all the variables on a causal structure are observed. Supposed that a causal structure contains a set of variables $V = \{V_1, ..., V_n\}$. The joint probability distribution can be expressed using the Markov factorization:

$$p(v_1, ..., v_n) = \prod_{k=1}^{n} p(v_k|pa(v_k)), \tag{1}$$

where each variable V_k is only conditioned on its parents in the causal graph. Consider now that we want to calculate the postinterventional distribution $p(x|do(Y = y))$, where X and Y are two disjoint subsets of V. Intervening $do(Y = y)$ is equivalent to removing the parents of all the variables $v_k \in Y$, since $p(Y = y'|do(Y = y)) = \delta_{yy'}$. Therefore the postinterventional joint probability distribution resulting from the intervention on $Y = y$ can be calculated from the observed joint distribution $p(V)$. In the Markov factorization of Eq. 1 the factors $\prod_{\{v_k \in Y\}} p(v_k|pa(v_k))$ are substituted by 1 if $Y = y$ and 0 if $Y \neq y$ resulting in:

$$p(V|do(Y = y)) = \begin{cases} \prod_{\{v_k \in V \setminus Y\}} p(v_k|pa(v_k)) & \text{if } Y = y \\ 0 & \text{if } Y \neq y, \end{cases} \tag{2}$$

that is, for $Y = y$

$$p(v|do(Y = y)) = \frac{p(v)}{\prod_{\{v_k \in Y\}} p(v_k|pa(v_k))}. \tag{3}$$

The postinterventional distribution of Eqs. 2 and 3 allows us to calculate the causal effect $p(x|do(Y = y))$ by marginalization of the joint postinterventional probability distribution. In particular

$$p(x|do(Y = y)) = \sum_{v_k \in V \setminus \{XY\}} \prod_{\{v_k \in V \setminus Y\}} p(v_k|pa(v_k)). \tag{4}$$

The procedure above is also applicable to calculate conditional causal effects given that

$$p(x|z, do(Y = y)) \triangleq \frac{p(x, z|do(Y = y))}{p(z|do(Y = y))}. \tag{5}$$

S2 Exemplary calculations of causal effects

Here we apply the procedure described above to calculate the causal effect $p(x|do(Y = y))$ and the conditional causal effect $p(x|Z = z, do(Y = y))$ for the two exemplary causal graphs of Figure S1A and S1B. We start considering the causal graph of Figure S1A. In that case the Markov factorization of the joint distribution is:

$$p(x, y, z) = p(x|y, z)p(y|z)p(z). \tag{6}$$

The postinterventional joint distribution is:

$$p(x, y, z|do(Y = y)) = p(x|y, z)p(z) \tag{7}$$

and the causal effect of $do(Y = y)$ on X is:

$$p(x|do(Y = y)) = \sum_z p(x|y, z)p(z). \tag{8}$$

Similarly the causal effect of $do(Y = y)$ on Z is:

$$p(z|do(Y = y)) = \sum_x p(x|y, z)p(z) = p(z), \tag{9}$$

and thus the conditional causal effect given $Z = z$ of $do(Y = y)$ on X is:

$$p(x|Z = z, do(Y = y)) = \frac{p(x, z|do(Y = y))}{p(z|do(Y = y))} = \frac{p(x|y, z)p(z)}{p(z)} = p(x|y, z). \tag{10}$$

Therefore we see that while the causal effect $p(x|do(Y = y))$ (Eq. 8) is not a natural causal effect (see Results section for a definition), the conditional causal effect $p(x|Z = z, do(Y = y))$ (Eq. 10) is a natural causal effect.

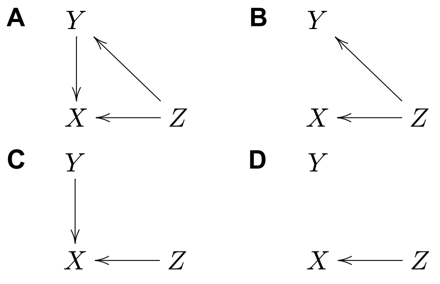

Figure S1: **Causal graphs illustrating the effect of interventions.** **A**: Graph showing a case where the statistical dependence between Y and X is (partly) due to a causal interaction from Y to X. **B**: Graph showing a case where the statistical dependence between Y and X is induced solely by the confounding variable Z. **C**: Graph corresponding to the intervention $do(Y = y')$ in the causal graph shown in **A**. **D**: Graph corresponding to the intervention $do(Y = y')$ in the causal graph shown in **B**.

We now turn to Figure S1B. The Markov factorization is:

$$p(x, y, z) = p(x|z)p(y|z)p(z). \tag{11}$$

The postinterventional joint distribution is:

$$p(x, y, z|do(Y = y)) = p(x|z)p(z) \tag{12}$$

and the causal effect of $do(Y=y)$ on X is:

$$p(x|do(Y=y)) = \sum_z p(x|z)p(z) = p(x). \tag{13}$$

Similarly the causal effect of $do(Y=y)$ on Z is:

$$p(z|do(Y=y)) = \sum_x p(x|z)p(z) = p(z), \tag{14}$$

and thus the conditional causal effect given $Z=z$ of $do(Y=y)$ on X is:

$$p(x|Z=z, do(Y=y)) = \frac{p(x,z|do(Y=y))}{p(z|do(Y=y))} = \frac{p(x|z)p(z)}{p(z)} = p(x|z). \tag{15}$$

In this case none of the causal effects fulfills the condition of existence of natural causal effects. In particular in this case the postinterventional distributions consistently indicate that intervening Y has no effect on X, and thus not only the causal effect is not natural but there is no causal effect at all.

S3 Conditions for the existence of natural causal effects

We here provide a proof of which are the conditions on the causal structure for which natural causal effects exist. Our results constitute an extended and more formal proof of the arguments provided in [1] for the so called action-observation exchange rule of *do* Calculus. Consider that we have $V = \{Y, Z, X, W\}$ where Y, Z, W,and X can be sets of variables. We want to see the conditions for which

$$p(x|z, do(y)) = p(x|z, y). \tag{16}$$

For that purpose we consider:

$$p(x|z, do(y)) = \frac{\sum_{V\backslash\{Z,X,Y\}} p(v\backslash y|do(y))}{\sum_{V\backslash\{Z,Y\}} p(v\backslash y|do(y))} = \frac{\sum_{V\backslash\{Z,X,Y\}} \frac{p(v)}{\prod_{\{v_k\in Y\}} p(v_k|pa(v_k))}}{\sum_{V\backslash\{Z,Y\}} \frac{p(v)}{\prod_{\{v_k\in Y\}} p(v_k|pa(v_k))}} \tag{17}$$

where $v\backslash y$ means a particular value of the variables $V\backslash Y$. The first equality is a direct application of the definition of conditional intervention (Eq. 5), while the second is a direct application of the definition of intervention (Eq. 3).

Now we divide V into $\bigcup pa(Y) \cup \{Z, X, Y\}$ and the rest, to which we refer by O. Here $\bigcup pa(y)$ is the set formed by all the parents of $v_k \in Y$. We can write

$$p(x|z, do(y)) = \frac{\sum_{V\backslash\{Z,X,Y\}} \frac{p(o|\bigcup pa(y)\cup\{z,x,y\})p(\bigcup pa(y)\cup\{z,x,y\})}{\prod_{\{v_k\in Y\}} p(v_k|pa(v_k))}}{\sum_{V\backslash\{Z,Y\}} \frac{p(o|\bigcup pa(y)\cup\{z,x,y\})p(\bigcup pa(y)\cup\{z,x,y\})}{\prod_{\{v_k\in Y\}} p(v_k|pa(v_k))}}. \tag{18}$$

The sum of $p(o|\bigcup pa(y) \cup \{z, x, y\})$ over O normalizes to 1. So we have:

$$p(x|z, do(y)) = \frac{\sum_{\bigcup pa(y)\backslash\{Z,X\}} \frac{p(\bigcup pa(y)\cup\{z,x,y\})}{\prod_{\{v_k\in Y\}} p(v_k|pa(v_k))}}{\sum_{(\bigcup pa(y)\cup X)\backslash Z} \frac{p(\bigcup pa(y)\cup\{z,x,y\})}{\prod_{\{v_k\in Y\}} p(v_k|pa(v_k))}}. \tag{19}$$

we now use the chain rule to explicitly consider the conditional probabilities of X:

$$p(x|z, do(y)) = \frac{\sum_{\bigcup pa(Y)\backslash\{Z,X\}} \frac{p(x|\{\bigcup pa(y)\backslash x\}\cup\{z,y\})p(\{\bigcup pa(y)\backslash x\}\cup\{z,y\})}{\prod_{\{v_k\in Y\}} p(v_k|pa(v_k))}}{\sum_{(\bigcup pa(Y)\cup X)\backslash Z} \frac{p(x|\{\bigcup pa(y)\backslash x\}\cup\{z,y\})p(\{\bigcup pa(y)\backslash x\}\cup\{z,y\})}{\prod_{\{v_k\in Y\}} p(v_k|pa(v_k))}}. \tag{20}$$

We will now derive sufficient conditions for Eq. 16. If $p(x|\{\bigcup pa(y)\backslash x\} \cup \{z, y\}) = p(x|z, y)$ we can write:

$$p(x|z, do(y)) = \frac{p(x|z, y) \sum_{\bigcup pa(Y)\backslash\{Z,X\}} \frac{p(\{\bigcup pa(y)\backslash x\}\cup\{z,y\})}{\prod_{\{v_k\in Y\}} p(v_k|pa(v_k))}}{\sum_X p(x|z, y) \sum_{\bigcup pa(Y)\backslash\{Z,X\}} \frac{p(\{\bigcup pa(y)\backslash x\}\cup\{z,y\})}{\prod_{\{v_k\in Y\}} p(v_k|pa(v_k))}}. \tag{21}$$

and if $X \cap \bigcup pa(Y) = \emptyset$ we sum $\sum_X p(x|z, y) = 1$ to obtain:

$$p(x|z, do(y)) = \frac{p(x|z, y) \sum_{\bigcup pa(Y)\backslash Z} \frac{p(\bigcup pa(y)\cup\{z,y\})}{\prod_{\{v_k\in Y\}} p(v_k|pa(v_k))}}{\sum_{\bigcup pa(Y)\backslash Z} \frac{p(\bigcup pa(y)\cup\{z,y\})}{\prod_{\{v_k\in Y\}} p(v_k|pa(v_k))}} = p(x|z, y). \tag{22}$$

and it is then clear that Eq. 16 is accomplished.

Therefore:

$$(X \cap \bigcup pa(Y) = \emptyset) \wedge (p(x|\{\bigcup pa(y)\backslash x\} \cup \{z, y\}) = p(x|z, y)) \Rightarrow p(x|z, do(y)) = p(x|z, y). \tag{23}$$

This means that the conditional causal effect given Z of the intervention of Y on X is observable if Y and Z block all the back-door paths from Y to X (so that there are not common drivers), and if X is not a parent of Y.

We will now consider the fulfillment of the opposite implication. Alternatively we can examine if

$$\neg(X \cap \bigcup pa(Y) = \emptyset) \vee \neg(p(x|\{\bigcup pa(y)\backslash x\} \cup \{z, y\}) = p(x|z, y)) \Rightarrow \neg(p(x|z, do(y)) = p(x|z, y)) \quad (24)$$

holds.

Consider first that $p(x|\{\bigcup pa(y)\backslash x\} \cup \{z, y\}) = p(x|z, y)$ is fulfilled but that $X \cap \bigcup pa(Y) \neq \emptyset$. In particular we assume that for a subset $Y_x \subseteq Y$ for each $y_j \in Y_x$ there is a subset $X_{y_j} \subseteq X$ so that $x_i \in pa(y_j)$ if $x_i \in X_{y_j}$. Accordingly

$$\prod_{\{v_k \in Y\}} p(v_k|pa(v_k)) = \prod_{\{v_k \in Y \backslash Y_x\}} p(v_k|pa(v_k)) \prod_{\{v_k \in Y_x\}} p(v_k|X_{v_k}, pa(v_k)\backslash X_{v_k}). \quad (25)$$

If in this case Eq. 16 was fulfilled, given Eq. 21 we would have that:

$$\sum_{\bigcup pa(Y)\backslash\{Z,X\}} \frac{p(\{\bigcup pa(y)\backslash x\} \cup \{z, y\})}{\prod_{\{v_k \in Y\}} p(v_k|pa(v_k))} = \sum_X p(x|z, y) \sum_{\bigcup pa(Y)\backslash\{Z,X\}} \frac{p(\{\bigcup pa(y)\backslash x\} \cup \{z, y\})}{\prod_{\{v_k \in Y\}} p(v_k|pa(v_k))}. \quad (26)$$

The right-hand-side of this Equation is independent of X given the summation across X. Oppositely, the left-hand-side of the Equation is dependent of X through the factors of $v_k \in Y_x$ in Eq. 25, which have some $x_i \in pa(y_j)$. This means that the fulfillment of Eq. 16 is not compatible with $p(x|\{\bigcup pa(y)\backslash x\} \cup \{z, y\}) = p(x|z, y)$ and $X \cap \bigcup pa(Y) \neq \emptyset$.

We can extend the argument above to see that in general if $X \cap \bigcup pa(Y) \neq \emptyset$ then Eq. 16 is not fulfilled. The summation on $\bigcup(pa(Y) \cup X)\backslash Z$ in the denominator of Eq. 20 renders it a function only of Z, (we refer to it by $g(z)$). Assuming that $p(x|z, do(y)) = p(x|z, y)$ holds we would have:

$$p(x|z, y) = \frac{\sum_{\bigcup pa(Y)\backslash\{Z,X\}} \frac{p(x|\{\bigcup pa(y)\backslash x\}\cup\{z,y\}) p(\{\bigcup pa(y)\backslash x\}\cup\{z,y\})}{\prod_{\{v_k \in Y\}} p(v_k|pa(v_k))}}{g(z)}. \quad (27)$$

In the numerator X appears in $p(x|\{\bigcup pa(y)\backslash x\} \cup \{z, y\})$ but also it appears conditioning in the factors $\prod_{\{v_k \in Y_x\}} p(v_k|X_{v_k}, pa(v_k)\backslash X_{v_k})$ that are included in $\prod_{\{v_k \in Y\}} p(v_k|pa(v_k))$. This means that the right hand side of Equation 27 contains a term $p(x|\cdot)$ but also terms $p(\cdot|x, \cdot)$. Therefore the equality in Eq. 27 cannot be fulfilled for all values of X.

As the last case we now consider when $X \cap \bigcup pa(Y) = \emptyset$ but $p(x|\{\bigcup pa(y)\backslash x\} \cup \{z, y\}) \neq p(x|z, y)$. This completes the casuistics for $\neg(X \cap \bigcup pa(Y) = \emptyset) \vee \neg(p(x|\{\bigcup pa(y)\backslash x\} \cup \{z, y\}) = p(x|z, y))$ being fulfilled. In this case we have that $\sum_X p(x|\{\bigcup pa(y)\backslash x\} \cup \{z, y\}) = 1$ in the denominator of Eq. 20. If Eq. 16 was fulfilled we would have:

$$p(x|z, y) = \frac{\sum_{\bigcup pa(Y)\backslash\{Z,X\}} \frac{p(x|\{\bigcup pa(y)\backslash x\}\cup\{z,y\}) p(\{\bigcup pa(y)\backslash x\}\cup\{z,y\})}{\prod_{\{v_k \in Y\}} p(v_k|pa(v_k))}}{\sum_{\bigcup pa(Y)\backslash\{Z,X\}} \frac{p(\{\bigcup pa(y)\backslash x\}\cup\{z,y\})}{\prod_{\{v_k \in Y\}} p(v_k|pa(v_k))}}. \quad (28)$$

This Equation is of the form $a(x) \sum_i c_i = \sum_i b_i(x) c_i$, where $a(x) = p(x|z, y)$, $b_i(x) = p(x|\{\bigcup pa(y)\backslash x\} \cup \{z, y\})$, and $c_i = p(\{\bigcup pa(y)\backslash x\} \cup \{z, y\}) / \prod_{\{v_k \in Y\}} p(v_k|pa(v_k))$. Therefore it cannot be fulfilled for all x unless $b_i(x) = a(x)$.

Altogether we can state that:

$$p(x|z, do(y)) = p(x|z, y) \Leftrightarrow (X \cap \bigcup pa(Y) = \emptyset) \wedge (p(x|\{\bigcup pa(y)\backslash x\} \cup \{z, y\}) = p(x|z, y)). \quad (29)$$

S4 Analysis of causal effects in exemplary linear Gaussian stationary processes

We now study an example of linear Gaussian stationary processes. In this case the study of causality is simplified because the information theoretic measures depend only on first and second order moments (see Methods and [2]). In particular, the transfer entropy corresponds to the measure of causality proposed by Geweke (1982) [3] as shown in Barnett et al. (2009) [4]. We consider the following linear Gaussian autoregressive process:

$$\begin{aligned} x_{i+1} &= ax_i + by_i + \nu_x + \epsilon_{x,i+1} \\ y_{i+1} &= cy_i + \nu_y + \epsilon_{y,i+1}, \end{aligned} \tag{30}$$

where the innovations (ϵ) have zero mean and $\mathbb{E}[\epsilon_{x,i}\epsilon_{x,j}] = \sigma^2(\epsilon_x)\delta_{ij}$, $\mathbb{E}[\epsilon_{y,i}\epsilon_{y,j}] = \sigma^2(\epsilon_y)\delta_{ij}$ and $\mathbb{E}[\epsilon_{x,i}\epsilon_{y,j}] = 0\ \forall i, j$. A unidirectional causal interaction from $\mathcal{Y}$ to $\mathcal{X}$ exists for $b > 0$. Therefore b is the coupling parameter associated with the effective connectivity from $\mathcal{Y}$ to $\mathcal{X}$. We calculate the information theoretic measures analytically using 10 time lags to account for the past X^i and Y^i.

In Figure S2A-D we show some relevant information theoretic measures for the inference of causality and analysis of causal effects in dependence on the coefficients a and b, keeping the remaining parameters constant ($c = 0.8$, the variance of the innovations $\sigma^2(\epsilon_x) = \sigma^2(\epsilon_y) = 1$, and the levels $\nu_x = \nu_y = 0$ so that the mean of the process is zero). The values of a and b are chosen so that the bivariate process remains stationary. In Figure S2A we see that the transfer entropy $T_{\mathcal{Y}\to\mathcal{X}}$ depends only on b and is zero only for $b = 0$, so that it correctly indicates the existence of a causal connections. The transfer entropy from $\mathcal{X}$ to $\mathcal{Y}$ is always zero (Result not shown) consistently indicating the existence of unidirectional causality. That $T_{\mathcal{Y}\to\mathcal{X}}$ is independent of a can be understood given that it quantifies only the extra reduction in uncertainty once the statistical dependencies with the own past have already been accounted.

In Figure S2B we show the relative comparison of single natural causal effects following Eq. 12 of the Results section. In particular we compare the natural causal effect of intervening to $Y^i = \overrightarrow{0}$. Given the existence of unidirectional causality the probability distribution $p(x_{i+1}|Y^i = y^i)$ is associated with a natural causal effect, and we take as a reference the distribution $p_0(x_{i+1}|Y^i = y^i)$ obtained for $b = 0$ and $a = 0$. This means that the KL-divergence of Eq. 12 (Results) should not be considered as a measure of strength of the causal effect when $a \neq 0$, but simply as a measure of the relative difference of the natural causal effects, since also for any other value of a and $b = 0$ there is no causal connection from $\mathcal{Y}$ to $\mathcal{X}$. We see that, in contrast to the transfer entropy, this KL-divergence for the single intervention $Y^i = \overrightarrow{0}$ is much more dependent on a than on b. The natural causal effect significantly depends on how X_{i+1} is connected to its own past because this determines how the impact of the causal connections $Y_k \to X_{k+1}$ is accumulated. In Figure S2C we display the average KL-divergence corresponding to Eq. 13 (Results section). Here the relative difference of each possible natural causal effect is weighted according to its probability of occurrence, thus providing a measure of the average relative difference of all the natural causal effects of this type going on in the system. We see that, when instead of considering the single natural intervention $Y^i = \overrightarrow{0}$ all the interventions are averaged, the dependence in b is larger for high a.

Apart from comparing the natural causal effects for different configurations we can also examine the impact that changes in the coupling parameter have in other probability distributions not associated with natural causal effects. This is the type of analysis we proposed to complement the usual assessment of the gain in effective connectivity in the DCM approach with measures that captures the impact of these changes in some particular aspect of the dynamics. In Figure S2D we show the KL-divergence of Eq. 16 (Results). We can see that a change in the effective connectivity (related to b) has a different impact depending on the causal connections to the own past (a).

In Figures S2E-H we show the same measures as in Figure S2A-D but now in dependence on b and the mean μ_y of process $\mathcal{Y}$. We keep $c = a = 0.8$ and the variance of the innovations $\sigma^2(\epsilon_x) = \sigma^2(\epsilon_y) = 1$.

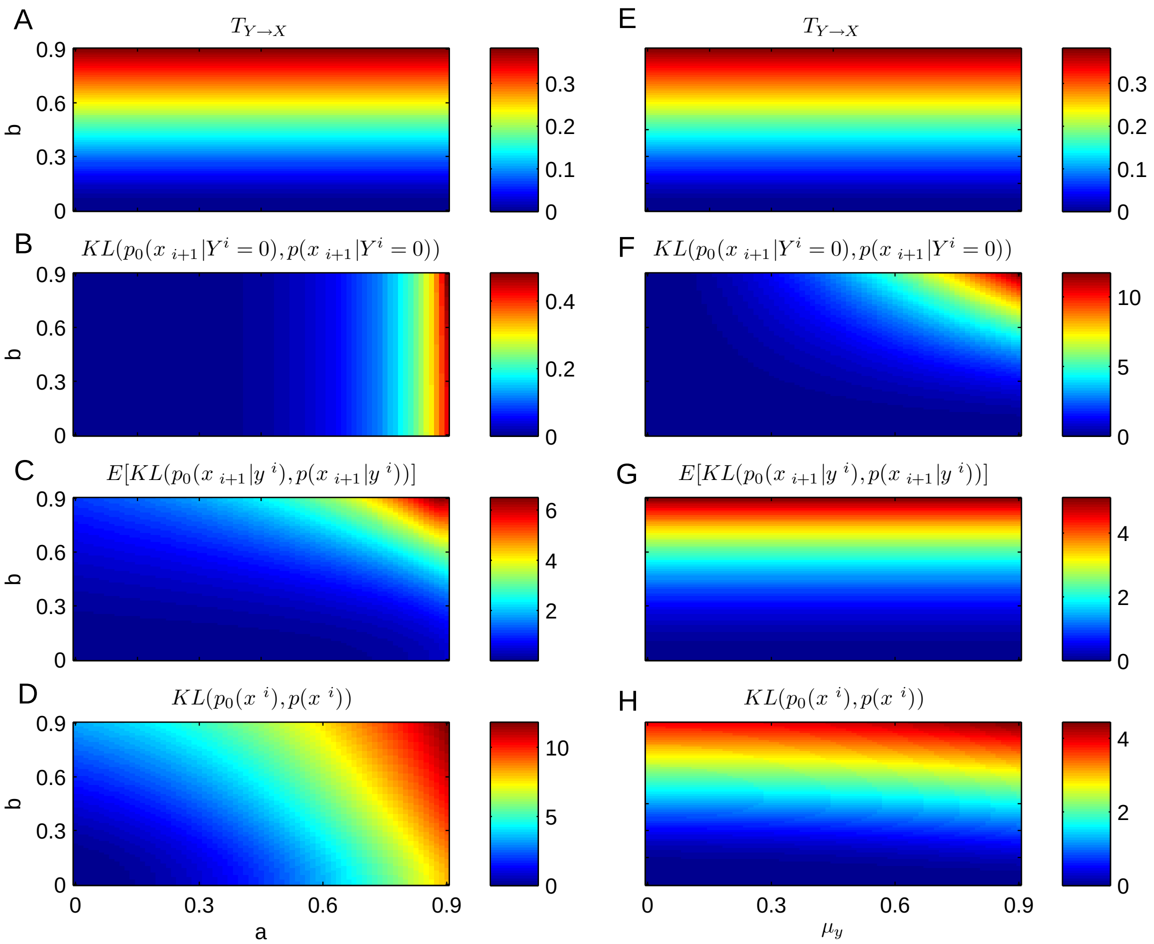

Figure S2: **Causality analysis in an AR(1) model.** Information theoretic measures used for the inference of causality and the analysis of causal effects calculated for a bivariate linear Gaussian stationary autoregressive process of order 1 (Equation 30). See the text for details about the processes. A-D: Dependence on b and a for $c = 0.8$, $\nu_x = \nu_y = 0$, and $\sigma^2(\epsilon_x) = \sigma^2(\epsilon_y) = 1$. E-H: Dependence on b and μ_y, the mean of the process $\mathcal{Y}$, for $c = a = 0.8$, $\sigma^2(\epsilon_x) = \sigma^2(\epsilon_y) = 1$, and $\nu_x = 0$.

We change the level ν_y to determine μ_y, and keep $\nu_x = 0$ so that the changes in the mean of $\mathcal{X}$ result only from the influence of $\mathcal{Y}$. We take as a reference the distribution for $b = 0$ and $\mu_y = 0$. In Figure S2E we show the transfer entropy $T_{\mathcal{Y} \to \mathcal{X}}$, which is also independent from μ_y. This results from the particular form of the entropy for Gaussian variables, which is completely determined by the variance [2] and independent of the mean.

In Figure S2F we compare again the natural causal effects resulting from the single natural intervention $Y^i = \overrightarrow{0}$. Given Eq. 22 (Methods section) it is clear that the KL-divergence of the single natural causal effects are sensitive to the mean, in contrast to the transfer entropy. Nonetheless, in this case this dependence is averaged out (Figure S2G) when considering the average difference of all the natural

causal effects. Like for the analysis of the dependence on b and a we here find that the dependence for particular single interventions (Figures S2B,F) can be very different from the average dependence (Figures S2C,G). As we explained above, our aim is not to introduce a complete set of measures to quantify causal effects, but rather to examine a framework to do so that is adaptive to the particular interests of each analysis.

Finally, in Figure S2H we show again the KL-divergence of Eq. 16 (Results). In this case we see that for the range of μ_y displayed, the impact of the change in effective connectivity is only slightly dependent on μ_y. These dependencies, like the ones displayed in Figure S2D are not easy to predict from a visual examination of the form of the model and from the gain in the coupling parameter, because they depend in a nonlinear way on a combination of several of the parameters and not only b.

References

1. Pearl J (1995) Causal diagrams for empirical research. Biometrika 82: 669-710.

2. Cover TM, Thomas JA (2006) Elements of Information Theory. John Wiley and Sons, 2nd edition.

3. Geweke JF (1982) Measurement of linear dependence and feedback between multiple time series. J Am Stat Assoc 77: 304-313.

4. Barnett L, Barrett AB, Seth AK (2009) Granger causality and transfer entropy are equivalent for gaussian variables. Phys Rev Lett 103: 238701.
